# Global burden of aortic aneurysm attributable to high-sodium diet from 1990 to 2021

**DOI:** 10.3389/fnut.2025.1653773

**Published:** 2025-08-21

**Authors:** Xiedong Shen, Weiling Tian, Chun Xue, Xiangguo Ji, Anqi Qu, Jun Lv

**Affiliations:** ^1^Department of Vascular and Endovascular Surgery, The Second Affiliated Hospital of Naval Medical University (Shanghai Changzheng Hospital), Shanghai, China; ^2^Interventional Radiology Department, The Second Affiliated Hospital of Naval Medical University (Shanghai Changzheng Hospital), Shanghai, China; ^3^Department of Gynecology and Obstetrics, The Second Affiliated Hospital of Naval Medical University (Shanghai Changzheng Hospital), Shanghai, China; ^4^School of Public Health, Southern Medical University, Foshan, Guangdong, China; ^5^Department of Emergency, The Second Affiliated Hospital of Naval Medical University (Shanghai Changzheng Hospital), Shanghai, China

**Keywords:** aortic aneurysm, diets high in sodium, global burden of disease, SDI, DALYs

## Abstract

**Background:**

This study aims to investigate the impact of high-sodium diet (HSD) on the global burden of aortic aneurysm (AA), analyze its epidemiological trends across different regions, sexes, and age groups, and project future trends.

**Methods:**

Data were obtained from the Global Burden of Disease Study 2021 (GBD 2021) to assess the disability-adjusted life years (DALYs) and death cases of AA attributable to HSD (AA-HSD) from 1990 to 2021. A decomposition analysis was conducted to quantify the contributions of population growth, aging, and epidemiological changes to the disease burden. The Nordpred model was used to project trends from 2022 to 2045.

**Results:**

From 1990 to 2021, the global DALYs cases of AA-HSD increased by 103%, and death cases increased by 120%. The largest increase in DALYs was observed in low-middle Socio-demographic Index (SDI) regions (234%), with South Asia experiencing the most significant rise (361%). Aging and population growth were the main drivers of the increased DALYs and death cases. While high SDI regions bore a heavier disease burden, relative inequality slightly improved. Projections indicate that from 2022 to 2045, both DALYs and death cases will continue to rise, with males experiencing a higher burden than females.

**Conclusion:**

HSD significantly contribute to the global burden of AA, particularly in low-middle SDI regions. Moving forward, differentiated strategies should be adopted based on regional development levels, such as reinforcing salt-reduction policies, promoting early screening programs.

## Introduction

Aortic aneurysm (AA) refers to an abnormal, localized dilation of the aortic lumen caused by degenerative changes or pathological injury to the aortic wall, with a diameter exceeding 1.5 times the normal value (1, 2). Based on the anatomical location, AA is classified into thoracic aortic aneurysms (TAA) and abdominal aortic aneurysms (AAA), with AAA being the most common (3–5). Although most AA are asymptomatic in the early stages, their rupture can lead to catastrophic consequences with extremely high mortality rates, making them of significant clinical and public health concern. The development of AA is associated with various factors, including congenital abnormalities, genetic disorders, atherosclerosis, infections, and trauma (1). Among these, atherosclerosis is the most common underlying cause. Moreover, certain hereditary conditions such as Marfan syndrome and Ehlers–Danlos syndrome significantly increase the risk of AA (6).

A high-sodium diet (HSD) is closely associated with AA (7). Epidemiological studies have identified it as one of the major risk factors for aneurysm-related mortality, with an even greater risk when combined with hypertension or smoking (8). Clinical research has demonstrated a positive correlation between high salt intake and the prevalence of AAA (9–11). In hypertensive animal models, a high-salt diet leads to elevated blood pressure and degradation of the aortic medial matrix, ultimately contributing to aneurysm formation. Nishijo et al. (9) found that transgenic mice developed thoracoabdominal aortic aneurysms after being administered high-salt solutions, with some experiencing aneurysm rupture during salt loading.

Although the harmful effects of a HSD on the cardiovascular system are well established, the global burden and regional disparities of AA attributable to HSD (AA-HSD) have not yet been quantified. This study, using data from the Global Burden of Disease Study 2021 (GBD 2021), is the first to systematically assess the trends in DALYs and deaths for AA-HSD. Through decomposition analysis, it reveals the relative contributions of population aging, growth, and epidemiological changes, and projects the disease burden through 2045, providing evidence to inform targeted salt reduction policies and early screening strategies.

## Methods

### Data sources and disease definition

The GBD 2021 database encompasses comprehensive data from 1990 to 2021, covering 204 countries and territories, 811 subnational regions, 371 diseases and injuries, and 88 risk factors (12, 13). The database provides a wide range of health metrics, including prevalence, incidence, mortality, and disability-adjusted life years (DALYs). The Socio-demographic Index (SDI), a composite indicator, is used to assess the level of socio-economic development and its association with health outcomes.

Moreover, the GBD 2021 database defined the optimal range of sodium intake as 1–5 g per day (measured by 24-h urinary sodium excretion), with a HSD defined as intake exceeding this upper limit (>5 g/day) (12). Data on dietary sodium intake were primarily derived from nationally representative dietary surveys, 24-h urinary sodium excretion studies, and published literature, including systematic reviews and meta-analyses (13). These data were compiled and standardized by the GBD team. To address data sparsity, measurement error, and heterogeneity across sources, the GBD study applied a Bayesian hierarchical meta-regression model (DisMod-MR 2.1) (12, 13). Adjustments were made for differences in survey methodologies, demographic distributions, and missing data through statistical imputation and calibration procedures. These processes ensured more accurate and comparable estimates of dietary sodium exposure across countries, years, and population groups.

AA refers to a localized dilation of the aortic wall, defined as an enlargement of the vessel diameter by more than 1.5 times the normal size. In the 10th Revision of the International Classification of Diseases (ICD-10), AA is coded as I71 (1). AA-HSD is defined as the burden of AA caused by actual sodium intake exceeding the theoretical minimum risk exposure level (>5 g/day) set by GBD 2021.

### Disability-adjusted life years

In the GBD 2021, DALYs are one of the core metrics used to measure disease burden, representing the combined impact of premature mortality and the decline in quality of healthy life caused by a specific disease or risk factor (14–16). DALYs are calculated as the sum of years of life lost due to premature death (YLLs) and years lived with disability (YLDs), using the formula: DALYs = YLLs + YLDs.

### Decomposition analysis

Decomposition analysis is a method used to precisely attribute changes or differences in disease burden metrics to various contributing factors (17, 18). In this study, we employed the Das Gupta decomposition method to quantify the contributions of population growth, aging, and epidemiological changes to the variation in disease burden. Furthermore, epidemiological changes refer to the changes in the incidence, prevalence, DALYs, or mortality of diseases or injuries after controlling for population size and age structure. They reflect the direct health effects brought about by improvements in medical services, preventive measures, changes in health behaviors, enhanced disease management, or other health interventions.

### Cross-country inequality analysis and prediction analysis

The Slope Index of Inequality (SII) is a key tool for measuring absolute inequality across countries, calculated by assessing the differences in health indicators between countries with the highest and lowest SDI values. The Concentration Index (CI) is used to evaluate the distribution of health metrics across countries at different SDI levels (19, 20). The Nordpred model, based on the age-period-cohort (APC) regression framework, was employed to project epidemiological trends (21).

## Results

### Global and regional burden of AA-HSD

**Figure 1:**
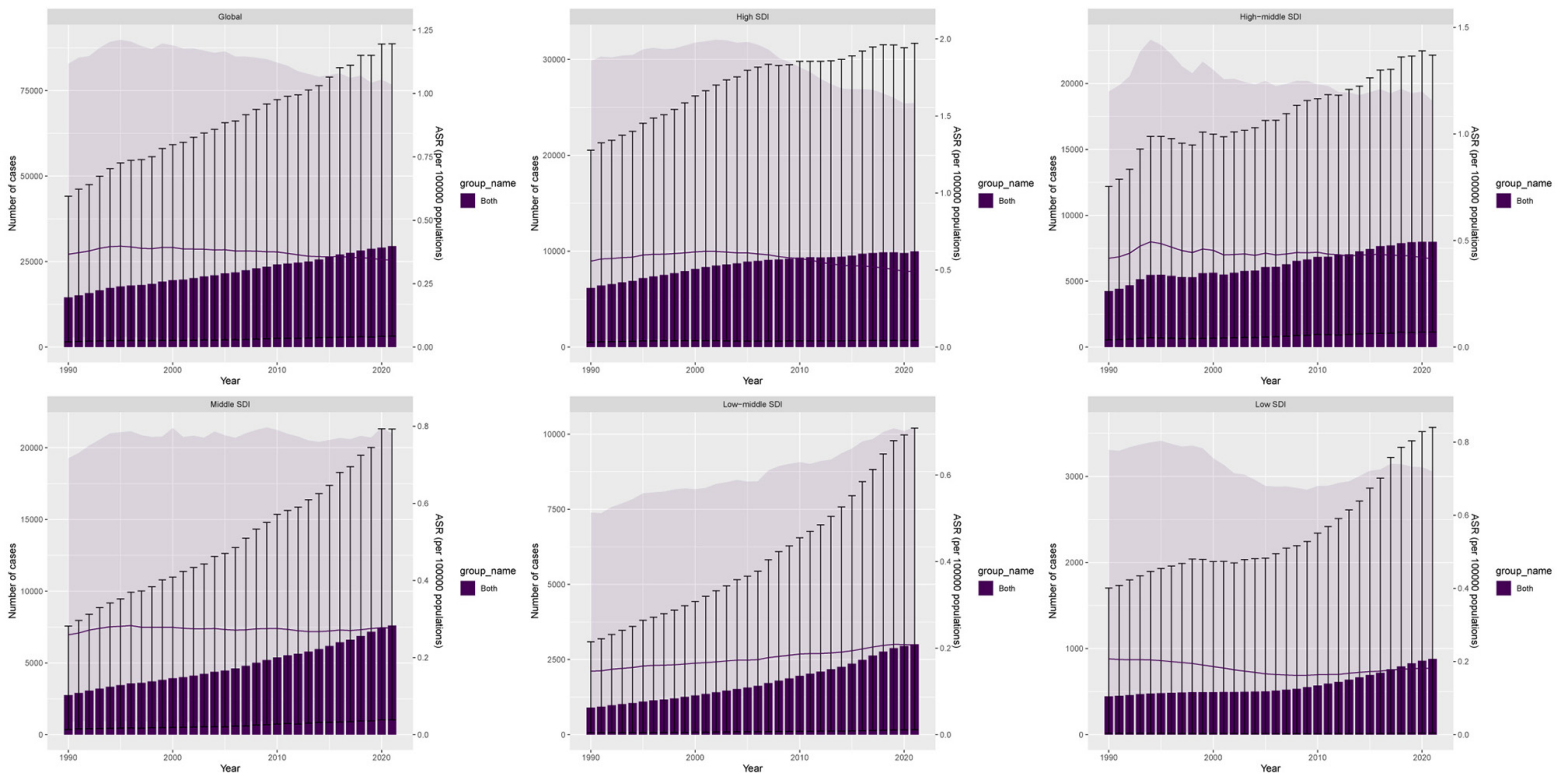
Disability-adjusted life years (DALYs) cases and ASDR of aortic aneurysm attributable to diet high in sodium from 1990 to 2021.

Over the past 32 years, the global number of DALYs cases of AA-HSD increased from 14,542.07 in 1990 to 29,507.29 in 2021, representing a 103% rise. The most significant increase was observed in low-middle SDI regions, where DALYs rose by 234%. Among the 21 GBD regions, South Asia experienced the largest percentage increase in DALYs, at 361%. In 2021, the global age-standardized death rate (ASDR) was 0.34 per 100,000 population, with an estimated annual percentage change (EAPC) of −0.38 (95% CI: −0.47 to −0.28). High SDI regions exhibited the highest ASDR at 0.49 per 100,000, with an EAPC of −0.56 (95% CI: −0.75 to −0.37). Conversely, low-middle SDI regions had the most rapid ASDR increase, with an EAPC of 1.14 (95% CI: 1.10 to 1.18) (Figure 1, Table 1).

**Table 1:** Disability-Adjusted Life Years (DALYs) and age-standardized DALY rate (ASDR) of aortic aneurysm attributable to diet high in sodium in 1990 and 2021, and the PC and EAPC from 1990 to 2021.

**Location**	**1990_DALYs cases (95% UI)**	**2021_DALYs cases (95% UI)**	**Percentage change**	**1990_ASDR_per 100,000 (95% UI)**	**2021_ASDR_per 100,000 (95% UI)**	**EAPC (95% CI)**
Andean Latin America	43.8 (0.99–143.41)	107.42 (2.36 to 380.64)	1.45	0.22 (0.01 to 0.73)	0.18 (0 to 0.65)	−0.61 (−0.73 to −0.49)
Australasia	111.24 (0.37 to 522.95)	90.95 (0.26 to 450.92)	−0.18	0.47 (0 to 2.2)	0.18 (0 to 0.84)	−3.51 (−3.65 to −3.38)
Caribbean	114.59 (0.69 to 448.05)	154.23 (0.21 to 618.96)	0.35	0.46 (0 to 1.78)	0.29 (0 to 1.15)	−1.98 (−2.17 to −1.79)
Central Asia	121.33 (9.9 to 357.85)	255.22 (10.48 to 885.23)	1.1	0.25 (0.02 to 0.73)	0.31 (0.01 to 1.07)	0.58 (0.32 to 0.83)
Central Europe	1,570.21 (289.08 to 3,839.91)	2,075.78 (363.15 to 5,076.6)	0.32	1.06 (0.2 to 2.61)	0.98 (0.17 to 2.39)	−0.47 (−0.58 to −0.37)
Central Latin America	370.87 (42.08 to 1,026.4)	838.02 (97.88 to 2,269.5)	1.26	0.44 (0.05 to 1.21)	0.33 (0.04 to 0.9)	−1.94 (−2.28 to −1.6)
Central Sub-Saharan Africa	23.47 (0 to 148.39)	61.58 (−0.01 to 376.17)	1.62	0.11 (0 to 0.7)	0.12 (0 to 0.74)	0.04 (−0.18 to 0.25)
East Asia	2,176.31 (428.46 to 4,914.73)	6,276.24 (1,180.19 to 14,861)	1.88	0.22 (0.04 to 0.51)	0.29 (0.05 to 0.71)	1.09 (0.93 to 1.25)
Eastern Europe	1,315.18 (75.01 to 4,212.69)	2,359.38 (133.13 to 7,447.82)	0.79	0.47 (0.03 to 1.51)	0.71 (0.04 to 2.25)	0.98 (0.67 to 1.28)
Eastern Sub-Saharan Africa	308.9 (16.5 to 1,023.78)	432.59 (12.7 to 1,711.61)	0.4	0.44 (0.02 to 1.44)	0.28 (0.01 to 1.07)	−1.95 (−2.17 to −1.72)
Global	14,542.07 (1,551.34 to 44,150.97)	29,507.29 (3,226.53 to 88,708.54)	1.03	0.37 (0.04 to 1.12)	0.34 (0.04 to 1.03)	−0.38 (−0.47 to −0.28)
High-income Asia Pacific	1,568.1 (284.25 to 3,635.22)	4,297.41 (310.56 to 12,632.48)	1.74	0.77 (0.14 to 1.8)	0.92 (0.07 to 2.69)	0.47 (0.29 to 0.64)
High-income North America	1,566.91 (3.63 to 6,804.15)	2,060.11 (38.1 to 7,545.51)	0.31	0.44 (0 to 1.94)	0.35 (0.01 to 1.24)	−1.14 (−1.51 to −0.77)
High-middle SDI	4,246.91 (550.33 to 12,191.53)	7,991.42 (1,136.98 to 22,154.1)	0.88	0.42 (0.05 to 1.2)	0.41 (0.06 to 1.15)	−0.23 (−0.36 to −0.1)
High SDI	6,160.31 (511.65 to 20,540.16)	9,984.21 (694.65 to 31,690.91)	0.62	0.56 (0.05 to 1.86)	0.49 (0.04 to 1.58)	−0.56 (−0.75 to −0.37)
Low-middle SDI	900.61 (62.36 to 3,094.93)	3,004.86 (170.36 to 10,202.35)	2.34	0.15 (0.01 to 0.51)	0.21 (0.01 to 0.71)	1.14 (1.1 to 1.18)
Low SDI	444.14 (15.76 to 1,703.79)	880.48 (17.84 to 3,568.86)	0.98	0.21 (0.01 to 0.78)	0.18 (0 to 0.72)	−0.62 (−0.85 to −0.4)
Middle SDI	2,756.83 (368.65 to 7,577.34)	7,603.78 (1,033.66 to 21,305.46)	1.76	0.26 (0.03 to 0.72)	0.28 (0.04 to 0.78)	−0.02 (−0.09 to 0.06)
North Africa and Middle East	62.16 (0.01 to 389.39)	204.27 (0.09 to 1,142.84)	2.29	0.03 (0 to 0.21)	0.04 (0 to 0.24)	0.9 (0.73 to 1.08)
Oceania	13.04 (0.95 to 40.72)	30.43 (2.02 to 98.04)	1.33	0.55 (0.04 to 1.62)	0.47 (0.03 to 1.46)	−0.87 (−1 to −0.75)
South Asia	626.14 (13.07 to 2,356.64)	2,888.01 (101.01 to 10,238.21)	3.61	0.11 (0 to 0.4)	0.19 (0.01 to 0.69)	2.12 (2.01 to 2.23)
Southeast Asia	814.95 (128.83 to 2,000.68)	2,073.98 (251.34 to 5,454.78)	1.54	0.34 (0.05 to 0.83)	0.33 (0.04 to 0.88)	−0.51 (−0.67 to −0.35)
Southern Latin America	456.68 (9.31 to 1,506.75)	445.63 (9.24 to 1,486.6)	−0.02	0.98 (0.02 to 3.23)	0.51 (0.01 to 1.71)	−2.34 (−2.53 to −2.15)
Southern Sub-Saharan Africa	60.05 (0.15 to 266.23)	85.83 (0.06 to 412.92)	0.43	0.21 (0 to 0.95)	0.14 (0 to 0.67)	−1.81 (−2.06 to −1.55)
Tropical Latin America	688.92 (29.95 to 2,287.02)	1,937.29 (60.2 to 6,467.12)	1.81	0.72 (0.03 to 2.37)	0.75 (0.02 to 2.48)	−0.29 (−0.54 to −0.05)
Western Europe	2,377.71 (49.85 to 9,182.65)	2,516.6 (43.32 to 9,802.02)	0.06	0.42 (0.01 to 1.6)	0.29 (0.01 to 1.07)	−1.4 (−1.6 to −1.19)
Western Sub-Saharan Africa	151.51 (−0.01 to 807.77)	316.33 (0.32 to 1,665.46)	1.09	0.18 (0 to 0.97)	0.16 (0 to 0.85)	−0.7 (−0.85 to −0.55)

Percentage change is expressed as a decimal, meaning the original value is multiplied by 100%.

Globally, death cases due to AA-HSD rose from 619.29 in 1990 to 1,362.64 in 2021, a 120% increase (Supplementary Figure 1). The sharpest growth in deaths occurred in low-middle SDI regions (251%). South Asia showed the largest increase in DALYs, with a 391% rise. In 2021, the global age-standardized mortality rate (ASMR) was 0.01625 per 100,000, with an EAPC of −0.27 (95% CI: −0.37 to −0.16). High SDI regions reported the highest ASMR at 0.02463 per 100,000, with an EAPC of −0.33 (95% CI: −0.53 to −0.14), while the greatest ASMR increase occurred in low-middle SDI regions, with an EAPC of 1.20 (95% CI: 1.16 to 1.24) (Table 2).

**Table 2:** Deaths and age-standardized mortality rate (ASMR) of aortic aneurysm attributable to diet high in sodium in 1990 and 2021, and the PC and EAPC from 1990 to 2021.

**Location**	**1990_Death cases (95% UI)**	**2021_Death cases (95% UI)**	**Percentage change**	**1990_ASMR_per 100,000 (95% UI)**	**2021_ASMR_per 100 000 (95% UI)**	**EAPC (95% CI)**
Andean Latin America	2.04 (0.05 to 6.8)	5.15 (0.11 to 17.99)	1.52	0.01107 (0.00027 to 0.03682)	0.00902 (0.00019 to 0.03127)	−0.66 (−0.78 to −0.54)
Australasia	5.3 (0.01 to 26.25)	4.98 (0 to 26.24)	−0.06	0.0224 (0.00004 to 0.11078)	0.00875 (0.00001 to 0.04529)	−3.39 (−3.56 to −3.22)
Caribbean	6.45 (0.05 to 24.8)	8.64 (0.02 to 34.63)	0.34	0.02717 (0.00023 to 0.10427)	0.0159 (0.00003 to 0.06382)	−2.24 (−2.44 to −2.05)
Central Asia	4.59 (0.39 to 13.22)	10.83 (0.47 to 36.31)	1.36	0.01009 (0.00085 to 0.0289)	0.01528 (0.00068 to 0.05005)	1.36 (1.04 to 1.68)
Central Europe	67.44 (12.28 to 161.55)	102.15 (17.65 to 251.29)	0.51	0.04711 (0.00856 to 0.11264)	0.04477 (0.00785 to 0.10965)	−0.36 (−0.46 to −0.27)
Central Latin America	15.26 (1.58 to 41.91)	37.11 (4.17 to 100.93)	1.43	0.01986 (0.00201 to 0.05558)	0.01522 (0.0017 to 0.04171)	−1.87 (−2.21 to −1.53)
Central Sub-Saharan Africa	0.94 (0 to 5.74)	2.47 (0 to 15.33)	1.63	0.00571 (0 to 0.03525)	0.00601 (0 to 0.0366)	0.02 (−0.21 to 0.25)
East Asia	69.8 (13.51 to 163)	221.43 (39.29 to 547.22)	2.17	0.00802 (0.00147 to 0.0191)	0.01034 (0.00178 to 0.02566)	0.99 (0.85 to 1.12)
Eastern Europe	47.57 (2.19 to 158.5)	92.33 (4.01 to 304.93)	0.94	0.01709 (0.00074 to 0.05717)	0.02666 (0.00127 to 0.08731)	1.09 (0.77 to 1.42)
Eastern Sub-Saharan Africa	13.32 (0.75 to 43.29)	19.21 (0.61 to 73.65)	0.44	0.02228 (0.0012 to 0.0708)	0.01444 (0.00048 to 0.0539)	−1.83 (−2.05 to −1.61)
Global	619.29 (58.61 to 1,923.49)	1,362.64 (126.8 to 4,149.57)	1.2	0.01696 (0.00149 to 0.05399)	0.01625 (0.00149 to 0.04962)	−0.27 (−0.37 to −0.16)
High-income Asia Pacific	72.35 (11.77 to 172.32)	279.74 (17.84 to 838.09)	2.87	0.03723 (0.00586 to 0.08944)	0.04901 (0.0035 to 0.14514)	0.86 (0.65 to 1.07)
High-income North America	77.87 (0.15 to 344.5)	94.32 (1.19 to 360)	0.21	0.02127 (0.00004 to 0.0934)	0.01444 (0.00023 to 0.05448)	−1.59 (−1.96 to −1.21)
High-middle SDI	160 (18.67 to 463.47)	321.76 (42.41 to 910.75)	1.01	0.01662 (0.00186 to 0.04853)	0.0164 (0.00218 to 0.04644)	−0.23 (−0.35 to −0.11)
High SDI	298.33 (21.72 to 1,038.11)	566.18 (34.38 to 1,838.14)	0.9	0.02648 (0.00193 to 0.09165)	0.02463 (0.0016 to 0.07871)	−0.33 (−0.53 to −0.14)
Low-middle SDI	35.73 (2.24 to 126.16)	125.5 (6.26 to 435.95)	2.51	0.00671 (0.00039 to 0.02405)	0.0096 (0.00044 to 0.03358)	1.2 (1.16 to 1.24)
Low SDI	18.85 (0.73 to 70.63)	37.76 (0.74 to 150.58)	1	0.01022 (0.00038 to 0.03828)	0.00905 (0.00018 to 0.0362)	−0.54 (−0.79 to −0.3)
Middle SDI	104.88 (13.44 to 294.42)	309.3 (39.45 to 877.12)	1.95	0.01162 (0.00129 to 0.0334)	0.01203 (0.00143 to 0.03426)	−0.15 (−0.24 to −0.07)
North Africa and Middle East	2.11 (0 to 13.26)	7.44 (0 to 43.94)	2.53	0.00125 (0 to 0.00816)	0.00169 (0 to 0.0105)	1.21 (1.02 to 1.39)
Oceania	0.6 (0.05 to 1.76)	1.37 (0.09 to 4.22)	1.28	0.03149 (0.00248 to 0.09103)	0.02517 (0.0017 to 0.07833)	−1.06 (−1.19 to −0.93)
South Asia	23.96 (0.38 to 91.92)	117.53 (3.49 to 428.86)	3.91	0.00474 (0.00006 to 0.01876)	0.00861 (0.00023 to 0.03215)	2.18 (2.06 to 2.31)
Southeast Asia	35.37 (5.27 to 87.81)	96.63 (10.59 to 257.74)	1.73	0.01683 (0.00233 to 0.04259)	0.01731 (0.00176 to 0.04709)	−0.37 (−0.51 to −0.22)
Southern Latin America	21.08 (0.41 to 69.44)	21.22 (0.41 to 71.27)	0.01	0.04655 (0.00091 to 0.15311)	0.02378 (0.00047 to 0.07997)	−2.42 (−2.61 to −2.23)
Southern Sub-Saharan Africa	2.21 (0 to 10.55)	3.17 (0 to 15.26)	0.43	0.00876 (0.00001 to 0.04337)	0.00593 (0 to 0.02935)	−1.81 (−2.09 to −1.54)
Tropical Latin America	26.69 (1.26 to 88.52)	84.32 (2.6 to 274.27)	2.16	0.03114 (0.00145 to 0.10351)	0.03331 (0.00102 to 0.10851)	−0.1 (−0.34 to 0.14)
Western Sub-Saharan Africa	6.73 (0 to 36.07)	13.22 (0.01 to 68.22)	0.96	0.00926 (0 to 0.0505)	0.0082 (0.00001 to 0.04253)	−0.81 (−0.98 to −0.65)

Percentage change is expressed as a decimal, meaning the original value is multiplied by 100%.

### National burden of AA-HSD

**Figure 2:**
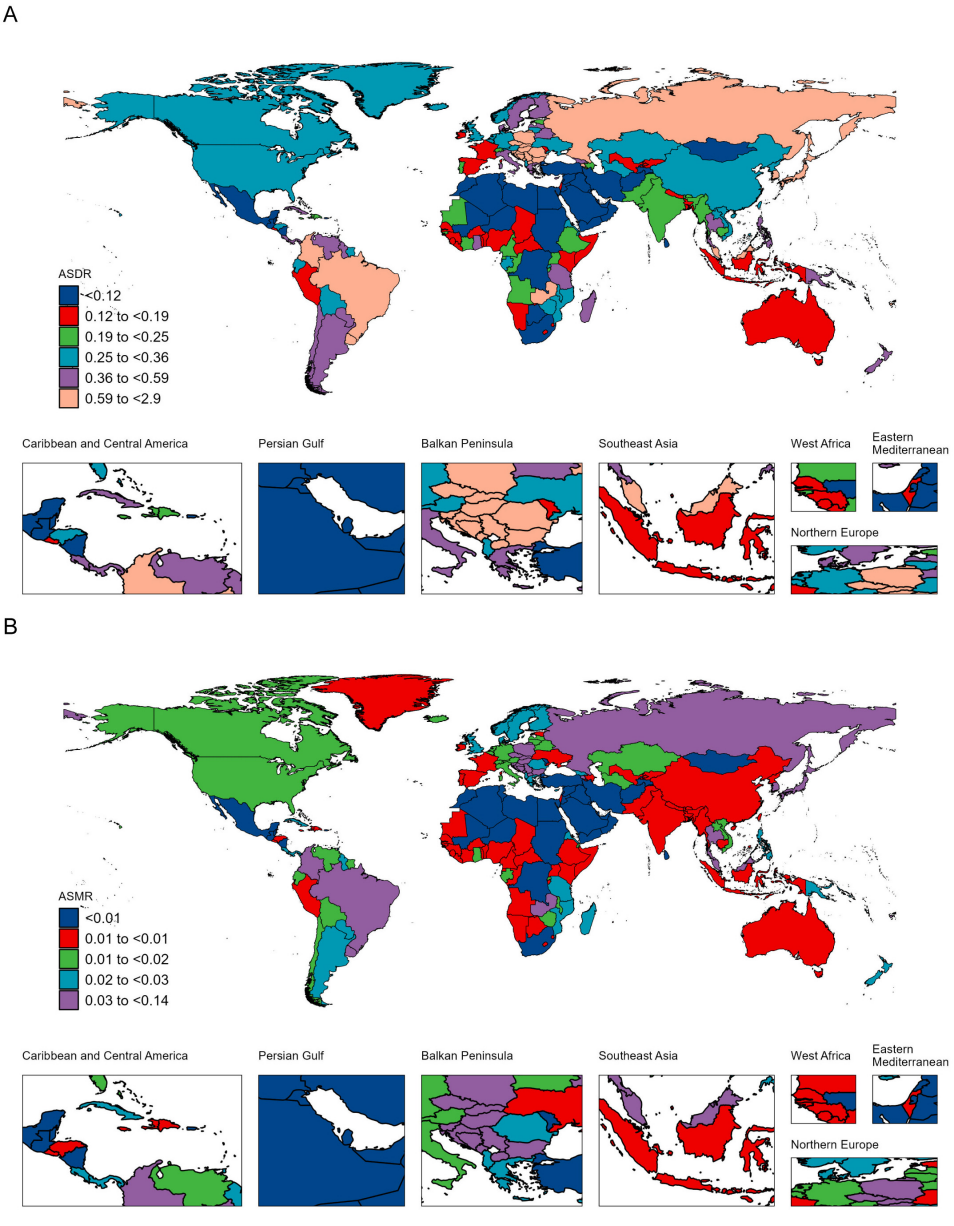
Age-standardized DALY rate (ASDR) **(A)** and age-standardized mortality rate (ASMR) **(B)** of aortic aneurysm attributable to diet high in sodium per 100,000 population in 2021, by country.

Between 1990 and 2021, the countries with the highest increases in DALYs were Saudi Arabia (1,046%), Oman (791%), and Yemen (744%). Georgia, Afghanistan, and Saudi Arabia had the most rapid ASDR increases, with EAPCs of 6.56 (95% CI: 5.20 to 7.94), 4.19 (95% CI: 3.92 to 4.46), and 4.18 (95% CI: 3.69 to 4.67), respectively (Figure 2A, Supplementary Table 1). The largest increases in death cases were observed in the United Arab Emirates (1,000%), Yemen (1,000%), and Saudi Arabia (800%). Georgia, Uzbekistan, and Morocco showed the fastest ASMR growth, with EAPCs of 6.68 (95% CI: 5.34 to 8.03), 4.46 (95% CI: 3.54 to 5.39), and 4.43 (95% CI: 4.18 to 4.67), respectively (Figure 2B, Supplementary Table 2).

### Age and sex differences in the burden of AA-HSD

**Figure 3:**
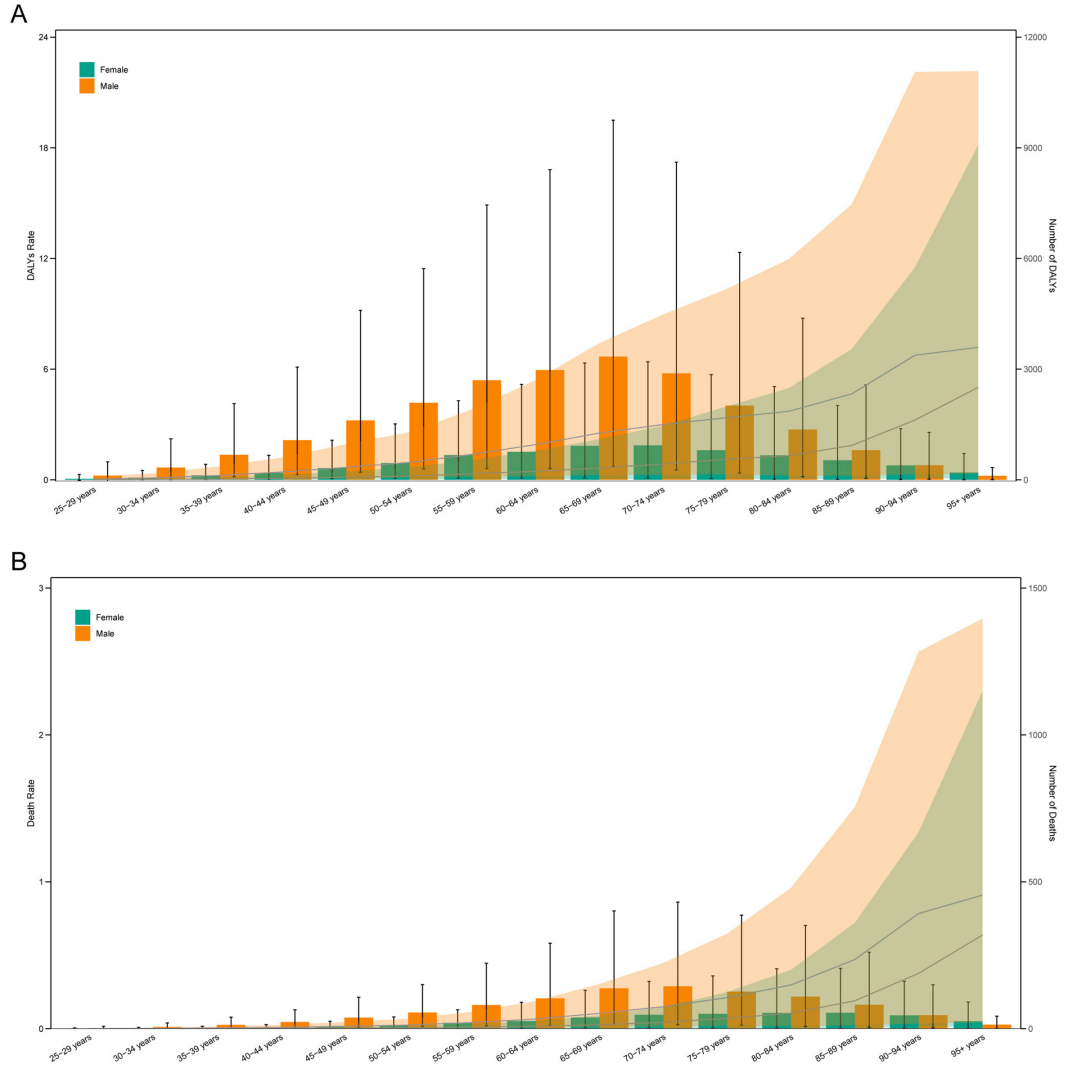
Age-specific numbers and rates of disability-adjusted life years (DALYs) **(A)** and deaths **(B)** of aortic aneurysm attributable to diet high in sodium by age and sex in 2021.

In 2021, males aged 65–69 had the highest number of DALYs (~3,338), while females aged 70–74 had the highest DALYs (~933). The highest number of deaths among males occurred in the 70–74 age group (~144), and among females in the 85–89 age group (~54). DALYs and death rates increased with age, peaking in the 95+ age group for both sexes (Figures 3A, B).

### Relationship between the burden of AA-HSD and SDI

**Figure 4:**
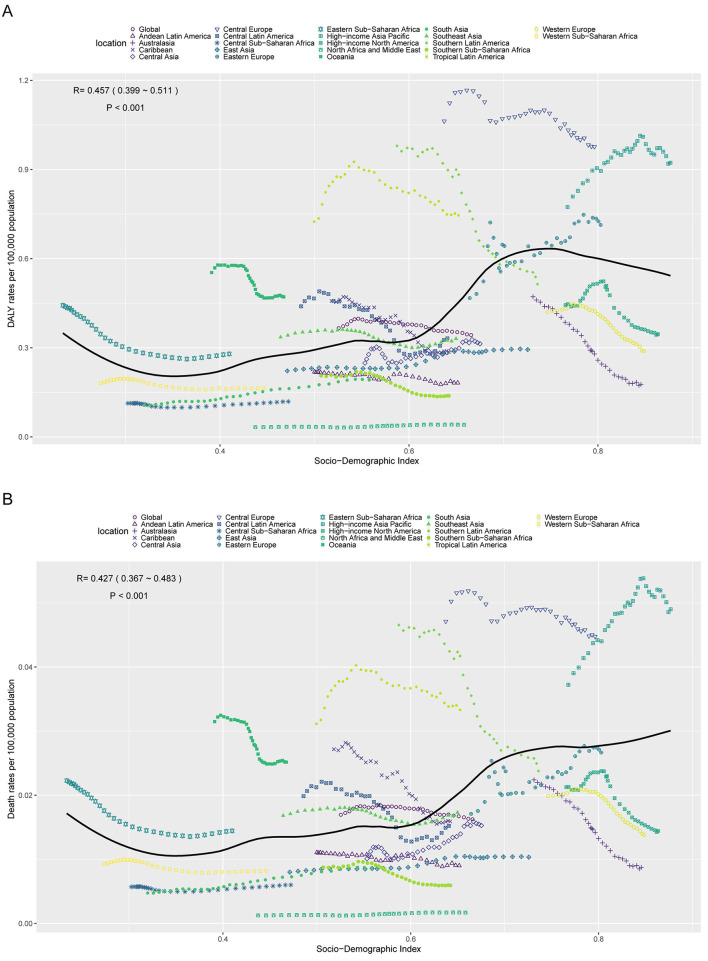
Age-standardized DALY rate (ASDR) **(A)** and age-standardized mortality rate (ASMR) **(B)** of aortic aneurysm attributable to diet high in sodium in 21 GBD regions by SDI, 1990–2021.

In 2021, ASDR and ASMR showed a significant positive correlation with SDI (Figures 4A, B). Specifically, ASDR increased with SDI levels between 0.3 and 0.75, but declined when SDI exceeded 0.75. In contrast, ASMR continued to increase with SDI levels beyond 0.3. Notably, some regions such as Oceania, Tropical Latin America, and Central Europe had a higher burden than expected, while others like North Africa and Middle East, Australasia, and Western Europe exhibited a lower-than-expected burden, warranting further investigation.

### Decomposition analysis of the burden of AA-HSD

**Figure 5:**
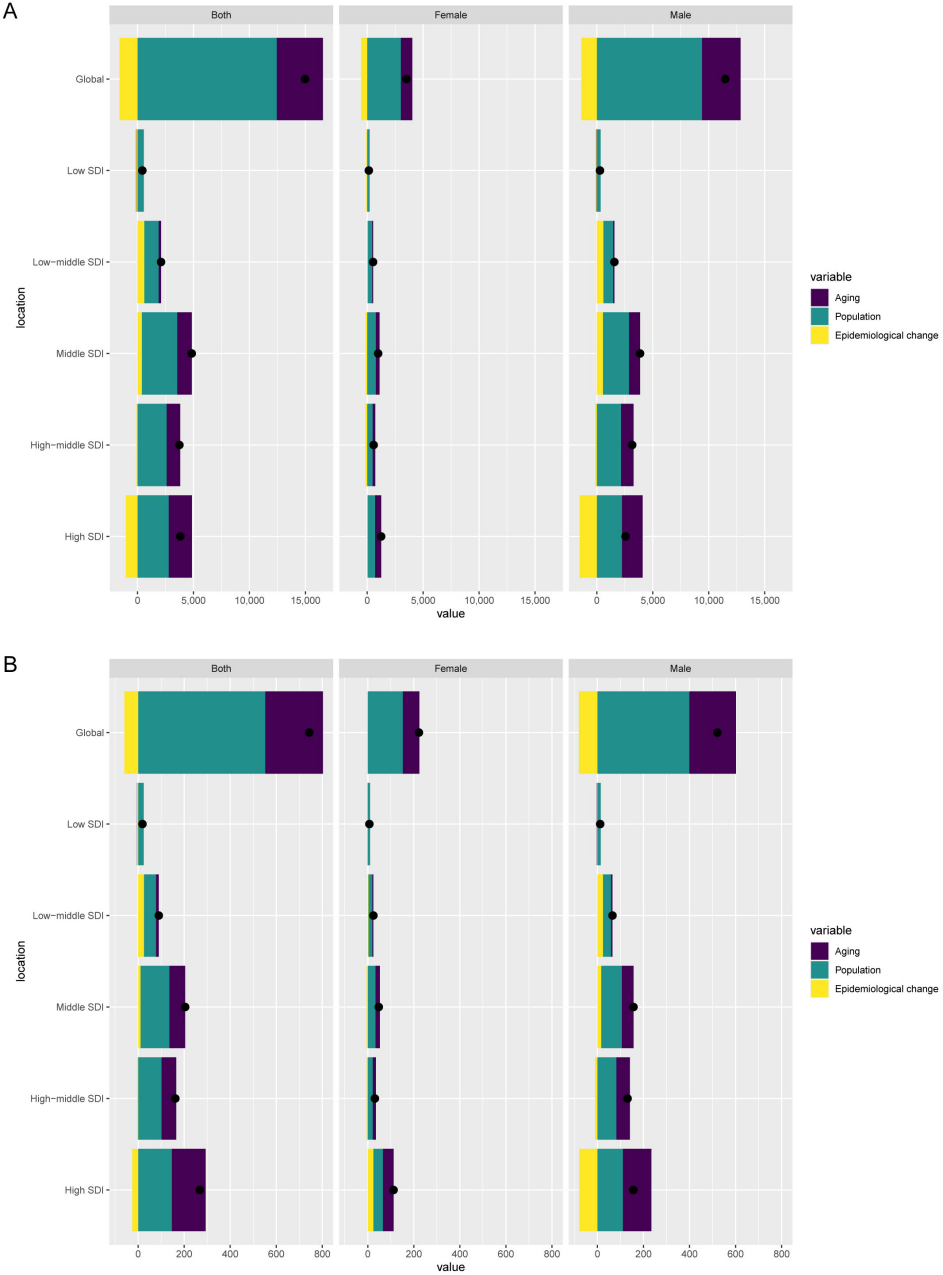
Decomposition analysis of changes in disability-adjusted life years (DALYs) **(A)** and deaths **(B)** of aortic aneurysm attributable to diet high in sodium between 1990 and 2021 across SDI regions.

Decomposition analysis revealed a total global increase of 14,965.22 DALYs from 1990 to 2021. Population aging contributed 4,128.01 DALYs (27.58%), population growth contributed 12,436.37 DALYs (83.10%), while epidemiological changes led to a reduction of 1,599.17 DALYs (−10.69%). Among males, DALYs increased by 11,460.54, with aging accounting for 3,445.01 (30.06%), population growth for 9,393.72 (81.97%), and epidemiological changes for −1,378.19 (−12.03%). Among females, the increase was 3,504.68 DALYs, with aging contributing 1,027.43 (29.32%), population growth contributing 3,005.00 (85.74%), and epidemiological changes contributing −527.76 (−15.06%) (Figure 5A).

Globally, deaths increased by 743.35 during the same period. Aging accounted for 250.79 (33.74%), population growth for 551.98 (74.26%), and epidemiological changes for −59.42 (−7.99%). Among males, deaths increased by 521.19, with aging contributing 202.71 (38.89%), population growth contributing 399.04 (76.56%), and epidemiological changes contributing −80.57 (−15.46%). Among females, the increase was 222.17 deaths, with aging accounting for 72.12 (32.46%), population growth for 152.08 (68.45%), and epidemiological changes for −2.03 (−0.91%) (Figure 5B).

### Cross-country inequality in the burden of AA-HSD

**Figure 6:**
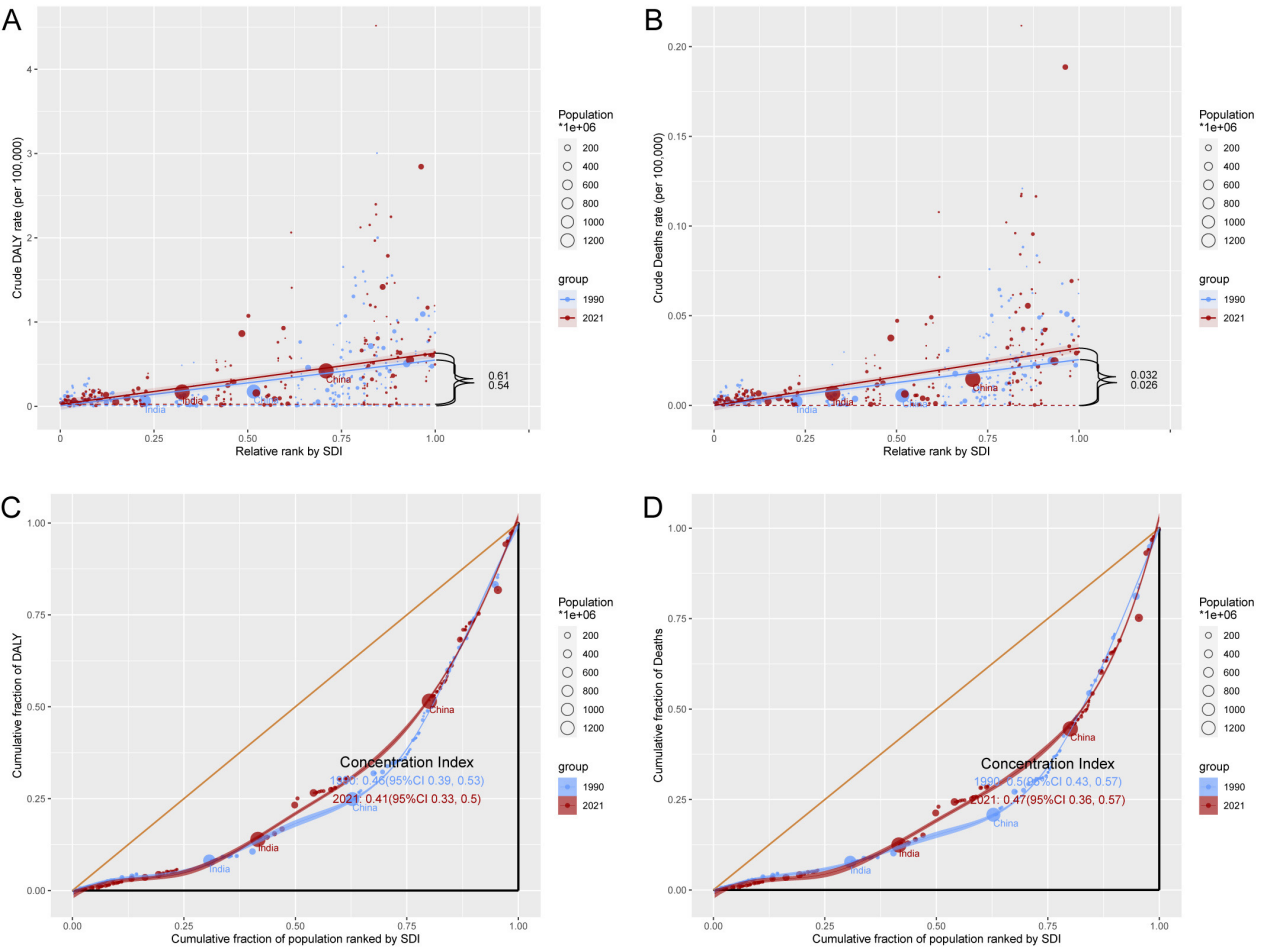
Inequality analysis of disability-adjusted life years (DALYs) and mortality in aortic aneurysm attributable to diet high in sodium in 1990 and 2021 across the world. **(A)** Health inequality regression curves for disability-adjusted life years (DALYs). **(B)** Health inequality regression curves for mortality. **(C)** Concentration curves for disability-adjusted life years (DALYs). **(D)** Concentration curves for mortality.

Between 1990 and 2021, higher SDI regions bore a disproportionately higher disease burden. The SII for DALY rates increased from 0.54 in 1990 to 0.61 in 2021, and for death rates from 0.026 to 0.032. However, the CI for DALY rates declined from 0.46 to 0.41, and for death rates from 0.50 to 0.47. This suggests that while the absolute disparity between high- and low-SDI regions widened, relative inequality across SDI regions improved slightly (Figures 6A-D).

### Forecasted burden of AA-HSD

**Figure 7:**
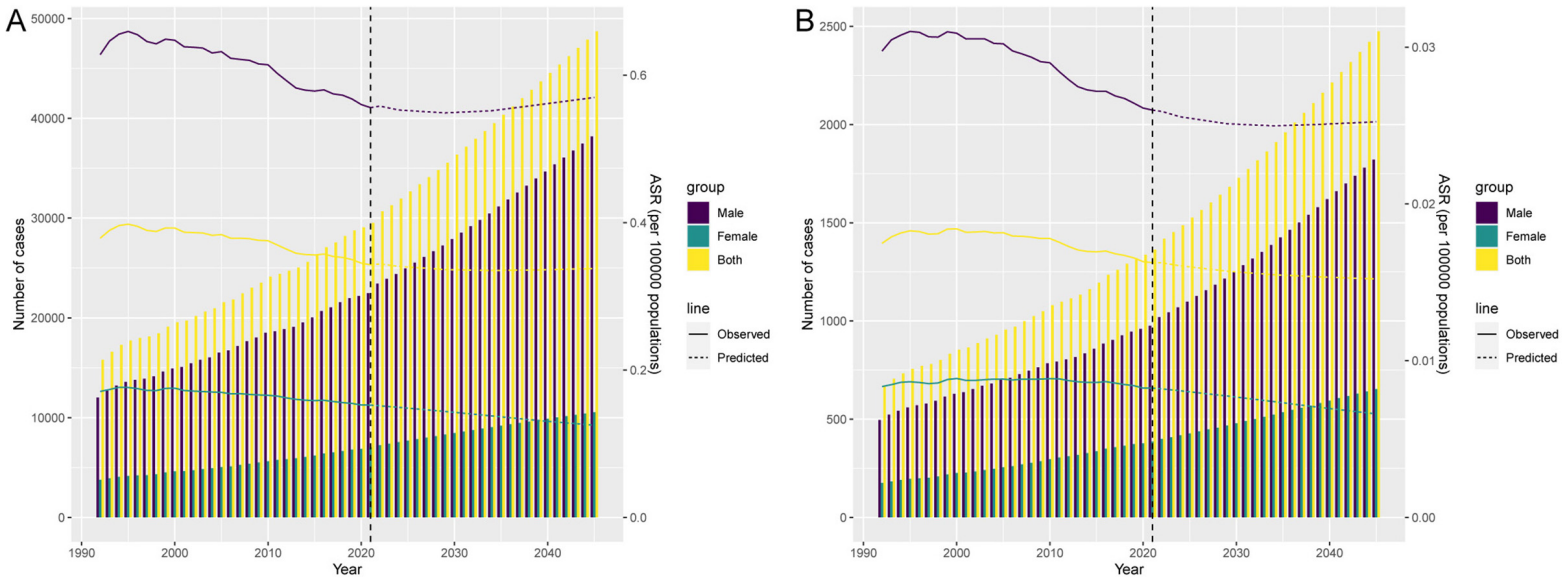
Projections of the temporal trends of the number of disability-adjusted life years (DALYs) cases, mortality cases, age-standardized DALY rate (ASDR), and age-standardized mortality rate (ASMR) of aortic aneurysm attributable to diet high in sodium globally up to 2045. **(A)** The number and age-standardized DALY rate (ASDR) of aortic aneurysm attributable to diet high in sodium by year and gender. **(B)** The number and age-standardized mortality rate (ASMR) of aortic aneurysm attributable to diet high in sodium by year and gender.

Forecast models predict a continued increase in DALYs and deaths of AA-HSD from 2022 to 2045, with a higher burden in males than females. By 2045, male DALYs are expected to reach approximately 38,176, while female DALYs will be around 10,536. Male deaths are projected to be 1,821 compared to 653 for females. Similarly, male ASDR is projected to reach 0.57 per 100,000 and female ASDR 0.13 per 100,000. Male ASMR is forecasted at 0.025 per 100,000, while female ASMR is expected to be 0.007 per 100,000 (Figures 7A, B).

## Discussion

Over the past 32 years, the global burden of AA-HSD has increased substantially, with DALYs and death cases rising by 103% and 120%, respectively. The most significant increases were observed in low-middle SDI regions, with South Asia experiencing the fastest-growing burden. Although the global ASMR showed a slight decline, high SDI regions continued to exhibit the highest mortality rates, while the fastest increases in ASMR occurred in low-middle SDI regions. This trend reflects the persistent impact of population aging and dietary risk factors, while also highlighting regional disparities. High SDI regions have benefited from advances in medical technology and preventive measures, contributing to a significant slowdown in the growth of death cases, whereas low-middle SDI regions face severe challenges due to dietary westernization and limited healthcare resources (22–24). Tailored strategies are needed, such as strengthening salt reduction policies, promoting early screening programs, and further investigating the interaction between high sodium intake and other risk factors.

In recent years, the burden of AA-HSD has grown significantly worldwide, particularly in certain countries and regions. Middle Eastern nations such as Saudi Arabia, Oman, and Yemen have seen the fastest increases in disease burden, likely due to dietary westernization and increased consumption of high-salt processed foods (22–24). Countries like Georgia, Uzbekistan, and Morocco exhibited the highest growth in ASMR, reflecting insufficient hypertension control and healthcare infrastructure in these areas. The elderly population, particularly males aged 65 and above, bears the greatest burden, with DALY and death rates significantly higher than those in females. This disparity may be due to men's greater exposure to cardiovascular risk factors (e.g., smoking, hypertension) and the protective effects of estrogen in women (25).

From 1990 to 2021, global DALYs and death cases of AA-HSD continued to increase, primarily driven by population growth and aging. However, this trend was partially mitigated by epidemiological changes, such as improved prevention and dietary modifications. Males consistently experienced a significantly higher burden than females. Cross-country inequality analysis revealed that while high SDI regions carry a heavier absolute burden, relative inequality has slightly improved. Projections suggest that from 2022 to 2045, DALYs and death cases will further rise, with males continuing to bear a disproportionately high burden, underscoring persistent sex-based disparities. This trend indicates the need for targeted interventions aimed at high-risk populations (e.g., males and residents of regions with high sodium intake), and the formulation of effective global sodium-reduction policies to address the health challenges posed by population aging and dietary risks (26).

In addition, from a pathophysiological perspective, excessive sodium intake induces sustained hypertension by activating the renin-angiotensin-aldosterone system (RAAS) and sympathetic nervous pathways, thereby increasing mechanical stress on the aortic wall, leading to vascular smooth muscle cell apoptosis and extracellular matrix degradation (9, 11). Animal models have confirmed that angiotensin II transgenic mice fed a high-salt diet can develop thoracoabdominal aortic aneurysms within a short period, characterized by elastin degradation and macrophage infiltration (11). Furthermore, a high-sodium environment induces NADPH oxidase-mediated oxidative stress, upregulates pro-inflammatory factors such as MMP-2/9, VCAM-1, CCL2, and TNF-α, directly damaging the elastin-collagen network and accelerating arterial dilation and aneurysmal remodeling (11).

This study has several limitations. First, the accuracy and completeness of data from the GBD 2021 may be influenced by the quality and methodology of the original data sources. Some countries and regions may lack reliable health surveillance systems, resulting in data biases or incompleteness. Second, data updates in the GBD 2021 database may have a degree of time lag, potentially failing to capture recent dietary trends and the latest effects of health interventions on AA burden in a timely manner.

## Conclusion

Over the past 32 years, the global burden of AA-HSD has increased markedly, with DALYs and death cases rising by 103% and 120%, respectively. South Asia emerged as the region with the fastest-growing burden. Saudi Arabia, Oman, and Yemen experienced the most significant increases in DALYs, while Georgia, Afghanistan, and Saudi Arabia showed the highest ASDR growth rates. Population aging and growth were the main drivers of the increase in DALYs and deaths, although epidemiological changes partially offset this trend. High SDI regions bore a heavier disease burden, but relative inequality showed slight improvement. Projections indicate that DALYs and deaths will continue to rise from 2022 to 2045, with men facing a higher burden than women. Therefore, region-specific strategies are urgently needed, such as reinforcing salt reduction initiatives, promoting early screening, and further exploring the interactions between high sodium intake and other risk factors to effectively address this growing global health challenge.

## Data Availability

The original contributions presented in the study are included in the article/Supplementary material, further inquiries can be directed to the corresponding authors.
