# Correction: Global burden of aortic aneurysm attributable to high-sodium diet from 1990 to 2021

**DOI:** 10.3389/fnut.2025.1700025

**Published:** 2025-09-25

**Authors:** Xiedong Shen, Weiling Tian, Chun Xue, Xiangguo Ji, Anqi Qu, Jun Lv

**Affiliations:** ^1^Department of Vascular and Endovascular Surgery, The Second Affiliated Hospital of Naval Medical University (Shanghai Changzheng Hospital), Shanghai, China; ^2^Interventional Radiology Department, The Second Affiliated Hospital of Naval Medical University (Shanghai Changzheng Hospital), Shanghai, China; ^3^Department of Gynecology and Obstetrics, The Second Affiliated Hospital of Naval Medical University (Shanghai Changzheng Hospital), Shanghai, China; ^4^School of Public Health, Southern Medical University, Foshan, Guangdong, China; ^5^Department of Emergency, The Second Affiliated Hospital of Naval Medical University (Shanghai Changzheng Hospital), Shanghai, China

**Keywords:** aortic aneurysm, diets high in sodium, global burden of disease, SDI, DALYs

In the published article, there was an error in the **Affiliations**. The institution for affiliation 1, 2, 3, and 5 were incorrectly written as “The Second Affiliated Hospital of Naval University”. The correct institution name for these affiliations should have been written as, “The Second Affiliated Hospital of Naval Medical University (Shanghai Changzheng Hospital).”

In the published article, there was an error in one of the author's names. The name of the author was incorrectly spelled as “Xiangfuo Ji”. The correct spelling should have been written as, “Xiangguo Ji”.

The original version of this article has been updated.

