# Supplementary material for: Global burden of aortic aneurysm attributable to high-sodium diet from 1990 to 2021
Source: Front Nutr. 2025 Aug 21;12:1653773. doi: 10.3389/fnut.2025.1653773 (PMC12408317; doi:10.3389/fnut.2025.1653773)
Supplement: Supplementary file 1 [file Table_1.docx]

| Location | 1990_DALYs cases (95% UI) | 2021_DALYs cases (95% UI) | Percentage change | 1990_ASDR_per 100000(95% UI) | 2021_ASDR_per 100 000(95% UI) | EAPC (95% CI) |
| --- | --- | --- | --- | --- | --- | --- |
| Afghanistan | 0.41 (0-2.52) | 2.06 (0-10.63) | 4.02 | 0.01 (0-0.04) | 0.02 (0-0.1) | 4.19 (3.92-4.46) |
| Albania | 7.67 (1.59-18.22) | 15.18 (2.44-41.52) | 0.98 | 0.37 (0.08-0.87) | 0.35 (0.06-0.97) | -0.08 (-0.22-0.05) |
| Algeria | 1.16 (0-6.52) | 7.97 (0-42.07) | 5.87 | 0.01 (0-0.05) | 0.02 (0-0.11) | 3.51 (3.19-3.83) |
| American Samoa | 0.06 (0-0.28) | 0.11 (0-0.45) | 0.83 | 0.33 (0-1.45) | 0.27 (0-1.08) | -1.16 (-1.38--0.93) |
| Andorra | 0.29 (0-1.47) | 0.44 (0-2.16) | 0.52 | 0.5 (0-2.52) | 0.29 (0-1.37) | -1.58 (-1.83--1.34) |
| Angola | 5.34 (0-30.62) | 21.38 (-0.01-115.13) | 3 | 0.15 (0-0.83) | 0.2 (0-1.05) | 0.66 (0.55-0.76) |
| Antigua and Barbuda | 0.2 (0-0.83) | 0.21 (0-0.89) | 0.05 | 0.36 (0-1.49) | 0.21 (0-0.87) | -2.59 (-2.9--2.28) |
| Argentina | 354.64 (5.46-1194.37) | 301.35 (5.24-1044.88) | -0.15 | 1.09 (0.02-3.66) | 0.54 (0.01-1.88) | -2.52 (-2.73--2.31) |
| Armenia | 33.68 (2.53-98.38) | 61.78 (2.26-211.86) | 0.83 | 1.21 (0.09-3.5) | 1.48 (0.06-5.1) | 0.82 (0.36-1.28) |
| Australia | 76.25 (0.14-385.76) | 58.58 (0.06-307.43) | -0.23 | 0.39 (0-1.94) | 0.14 (0-0.69) | -3.62 (-3.76--3.49) |
| Austria | 59.99 (0.86-204.22) | 52.41 (1.26-166.6) | -0.13 | 0.51 (0.01-1.75) | 0.29 (0.01-0.92) | -1.97 (-2.17--1.78) |
| Azerbaijan | 10.79 (0.86-32.88) | 26.74 (0.64-106.18) | 1.48 | 0.21 (0.02-0.64) | 0.25 (0.01-0.95) | 0.9 (0.59-1.21) |
| Bahamas | 0.67 (0-2.8) | 1.16 (0-5.03) | 0.73 | 0.45 (0-1.84) | 0.3 (0-1.27) | -1.67 (-1.99--1.34) |
| Bahrain | 0.08 (0-0.41) | 0.41 (0-2.44) | 4.12 | 0.03 (0-0.19) | 0.03 (0-0.21) | -0.05 (-0.37-0.27) |
| Bangladesh | 39.22 (0-177.82) | 223.3 (1.35-884.47) | 4.69 | 0.09 (0-0.4) | 0.17 (0-0.67) | 2.63 (2.3-2.95) |
| Barbados | 0.69 (0-3.14) | 0.75 (0-3.62) | 0.09 | 0.23 (0-1.04) | 0.14 (0-0.69) | -2.3 (-2.64--1.96) |
| Belarus | 36.3 (0.03-154.89) | 56.56 (0.05-243.78) | 0.56 | 0.28 (0-1.21) | 0.38 (0-1.64) | 0.34 (-0.16-0.83) |
| Belgium | 154.92 (4.34-496.77) | 75.14 (1.16-265.44) | -0.51 | 0.98 (0.03-3.11) | 0.32 (0.01-1.11) | -4.06 (-4.24--3.87) |
| Belize | 0.1 (0-0.44) | 0.24 (0-1) | 1.4 | 0.11 (0-0.48) | 0.09 (0-0.35) | -1.65 (-2.4--0.9) |
| Benin | 3.08 (0-14.05) | 7.72 (0-38.3) | 1.51 | 0.16 (0-0.73) | 0.16 (0-0.76) | -0.23 (-0.32--0.14) |
| Bermuda | 0.84 (0-3.38) | 0.62 (0-2.48) | -0.26 | 1.38 (0-5.6) | 0.43 (0-1.73) | -3.94 (-4.09--3.78) |
| Bhutan | 0.18 (0-0.81) | 1.25 (0.01-4.98) | 5.94 | 0.08 (0-0.37) | 0.21 (0-0.85) | 3.78 (3.54-4.02) |
| Bolivia (Plurinational State of) | 8.37 (0.16-28.43) | 23.43 (0.37-83.32) | 1.8 | 0.27 (0.01-0.9) | 0.26 (0-0.93) | -0.18 (-0.27--0.09) |
| Bosnia and Herzegovina | 43.24 (7.31-95.53) | 70.13 (10.89-173.26) | 0.62 | 1.03 (0.18-2.22) | 1.14 (0.18-2.88) | 0.38 (0.23-0.53) |
| Botswana | 1.07 (0-5.16) | 1.85 (0-10.14) | 0.73 | 0.18 (0-0.9) | 0.12 (0-0.66) | -1.87 (-2.05--1.7) |
| Brazil | 678.86 (29.53-2255.61) | 1902.58 (58.24-6370.51) | 1.8 | 0.73 (0.03-2.39) | 0.75 (0.02-2.5) | -0.31 (-0.56--0.07) |
| Brunei Darussalam | 1.73 (0.2-4.67) | 4.8 (0.44-12.75) | 1.77 | 1.68 (0.18-4.54) | 1.37 (0.13-3.67) | -0.4 (-0.53--0.26) |
| Bulgaria | 124.35 (25.72-273.83) | 146.08 (27.15-360.16) | 0.17 | 1.05 (0.22-2.33) | 1.13 (0.21-2.77) | 0.04 (-0.18-0.26) |
| Burkina Faso | 5.77 (0-30.7) | 15.54 (0-74.24) | 1.69 | 0.14 (0-0.75) | 0.18 (0-0.86) | 1.03 (0.96-1.1) |
| Burundi | 13.48 (0.49-45.46) | 9.75 (0.19-42.01) | -0.28 | 0.6 (0.02-2) | 0.22 (0-0.97) | -3.88 (-4.28--3.47) |
| Cabo Verde | 0.32 (0-1.84) | 0.82 (0-4.22) | 1.56 | 0.14 (0-0.81) | 0.19 (0-0.95) | 0.47 (0.24-0.7) |
| Cambodia | 10.01 (1.44-26.13) | 27.75 (2.35-80.71) | 1.77 | 0.23 (0.03-0.61) | 0.23 (0.02-0.71) | -0.01 (-0.15-0.12) |
| Cameroon | 8.99 (-0.01-43.33) | 24.6 (0-112.47) | 1.74 | 0.22 (0-1.04) | 0.2 (0-0.88) | -0.69 (-0.93--0.45) |
| Canada | 280.02 (2.36-1041.28) | 229.44 (3.41-831.27) | -0.18 | 0.85 (0.01-3.15) | 0.34 (0.01-1.21) | -3.63 (-3.9--3.35) |
| Central African Republic | 1.52 (0-8.89) | 3.02 (0-17.19) | 0.99 | 0.15 (0-0.84) | 0.15 (0-0.81) | -0.27 (-0.43--0.11) |
| Chad | 3.15 (0-15.83) | 7.53 (-0.01-40.33) | 1.39 | 0.11 (0-0.59) | 0.14 (0-0.77) | 0.44 (0.33-0.55) |
| Chile | 50.08 (1.58-158.3) | 96.33 (2.36-325.62) | 0.92 | 0.49 (0.01-1.56) | 0.38 (0.01-1.28) | -0.84 (-1.06--0.63) |
| China | 2053.96 (403.82-4619.18) | 5977.53 (1156.09-14109.07) | 1.91 | 0.22 (0.04-0.5) | 0.29 (0.06-0.69) | 1.19 (0.99-1.39) |
| Colombia | 247.88 (35.33-639.84) | 526.55 (79.97-1258.9) | 1.12 | 1.37 (0.2-3.51) | 0.95 (0.14-2.28) | -2.54 (-2.98--2.1) |
| Comoros | 1.09 (0.04-3.97) | 1.42 (0.02-5.97) | 0.3 | 0.58 (0.02-2.05) | 0.31 (0-1.29) | -2.56 (-2.83--2.3) |
| Congo | 2.13 (0-12.14) | 5.5 (0-29.76) | 1.58 | 0.21 (0-1.16) | 0.22 (0-1.13) | -0.38 (-0.57--0.18) |
| Cook Islands | 0.1 (0.01-0.3) | 0.18 (0.01-0.62) | 0.8 | 0.93 (0.08-2.64) | 0.68 (0.04-2.35) | -1.32 (-1.44--1.2) |
| Costa Rica | 8.26 (0.5-24.86) | 27.91 (1.71-79.3) | 2.38 | 0.47 (0.03-1.4) | 0.51 (0.03-1.44) | -0.26 (-0.5--0.02) |
| C么te d'Ivoire | 8.85 (0-40.64) | 24.05 (0-117.49) | 1.72 | 0.24 (0-1.08) | 0.22 (0-1.06) | -0.77 (-0.96--0.57) |
| Croatia | 72.06 (13.95-162.01) | 95.81 (17.33-223.98) | 0.33 | 1.18 (0.23-2.61) | 1.11 (0.2-2.62) | -0.46 (-0.68--0.25) |
| Cuba | 72.71 (0.42-282.18) | 85.65 (0.1-351.3) | 0.18 | 0.71 (0-2.75) | 0.43 (0-1.77) | -2.17 (-2.42--1.93) |
| Cyprus | 5.11 (0.01-21.87) | 8.22 (0.04-32.88) | 0.61 | 0.69 (0-2.82) | 0.4 (0-1.57) | -2.02 (-2.2--1.83) |
| Czechia | 206.02 (40.53-463.63) | 239.16 (39.36-553.71) | 0.16 | 1.51 (0.29-3.4) | 1.13 (0.19-2.62) | -1.14 (-1.46--0.8) |
| Democratic People's Republic of Korea | 65.9 (11.75-158.48) | 90.05 (13.67-238.35) | 0.37 | 0.36 (0.06-0.84) | 0.27 (0.04-0.7) | -0.95 (-1.03--0.87) |
| Democratic Republic of the Congo | 12.86 (0-88.24) | 27.96 (0-202.78) | 1.17 | 0.09 (0-0.6) | 0.08 (0-0.58) | -0.3 (-0.69-0.1) |
| Denmark | 55.24 (0.28-213.5) | 48.27 (0.21-201.34) | -0.13 | 0.73 (0-2.79) | 0.41 (0-1.65) | -2.18 (-2.5--1.87) |
| Djibouti | 0.69 (0.03-2.33) | 1.64 (0.02-6.52) | 1.38 | 0.56 (0.02-1.84) | 0.29 (0-1.09) | -2.62 (-2.83--2.42) |
| Dominica | 0.25 (0-1.05) | 0.32 (0-1.28) | 0.28 | 0.42 (0-1.74) | 0.39 (0-1.57) | -0.67 (-0.87--0.48) |
| Dominican Republic | 6.08 (0.02-23.21) | 18.77 (0.02-76.97) | 2.09 | 0.18 (0-0.67) | 0.19 (0-0.78) | 0.01 (-0.17-0.2) |
| Ecuador | 15.7 (0.33-50.9) | 41.87 (0.73-148.7) | 1.67 | 0.3 (0.01-0.99) | 0.26 (0-0.92) | -0.23 (-0.57-0.11) |
| Egypt | 6.61 (0-36.62) | 19.06 (0-102.05) | 1.88 | 0.02 (0-0.12) | 0.03 (0-0.15) | 0.56 (0.46-0.66) |
| El Salvador | 4.07 (0.25-12.48) | 8.05 (0.49-24.7) | 0.98 | 0.14 (0.01-0.42) | 0.13 (0.01-0.4) | -0.31 (-0.49--0.13) |
| Equatorial Guinea | 0.27 (0-1.61) | 1.09 (0-6.48) | 3.04 | 0.15 (0-0.85) | 0.22 (0-1.17) | 1.01 (0.89-1.13) |
| Eritrea | 5.44 (0.17-19.7) | 8.16 (0.13-36.02) | 0.5 | 0.5 (0.02-1.73) | 0.34 (0.01-1.45) | -1.83 (-2.04--1.62) |
| Estonia | 3.3 (0-17.56) | 4.79 (0-25.11) | 0.45 | 0.16 (0-0.85) | 0.19 (0-1.02) | -0.13 (-0.51-0.26) |
| Eswatini | 0.53 (0-2.46) | 0.9 (0-4.22) | 0.7 | 0.18 (0-0.86) | 0.14 (0-0.7) | -0.78 (-1.05--0.51) |
| Ethiopia | 61.45 (2.93-209.11) | 79.42 (1.7-311.95) | 0.29 | 0.34 (0.02-1.13) | 0.2 (0-0.8) | -2.12 (-2.39--1.86) |
| Fiji | 2.44 (0.19-7.39) | 5.29 (0.37-15.62) | 1.17 | 0.85 (0.07-2.51) | 0.76 (0.05-2.25) | -0.92 (-1.17--0.67) |
| Finland | 50.67 (0.3-184.57) | 44.13 (0.44-174.99) | -0.13 | 0.75 (0.01-2.68) | 0.39 (0.01-1.45) | -2.08 (-2.42--1.74) |
| France | 240.34 (0.06-1093.87) | 241.15 (0.2-1031.33) | 0 | 0.29 (0-1.33) | 0.18 (0-0.74) | -1.9 (-2.19--1.62) |
| Gabon | 1.36 (0-7.44) | 2.61 (0-14.51) | 0.92 | 0.25 (0-1.37) | 0.27 (0-1.45) | -0.15 (-0.34-0.03) |
| Gambia | 0.59 (0-3.16) | 1.9 (0-9.65) | 2.22 | 0.17 (0-0.92) | 0.2 (0-1.01) | 0.07 (-0.09-0.23) |
| Georgia | 8.36 (0.71-24.11) | 26.81 (0.96-88.96) | 2.21 | 0.14 (0.01-0.39) | 0.49 (0.02-1.62) | 6.56 (5.2-7.94) |
| Germany | 379.47 (3.45-1376.31) | 515.55 (7.25-1970.4) | 0.36 | 0.31 (0-1.1) | 0.32 (0.01-1.15) | 0.25 (0.15-0.34) |
| Ghana | 18.65 (0-90.03) | 60.81 (0.22-269.39) | 2.26 | 0.31 (0-1.47) | 0.36 (0-1.61) | 0.51 (0.34-0.68) |
| Greece | 62.12 (0.35-239.19) | 119.99 (0.75-488.08) | 0.93 | 0.42 (0-1.62) | 0.57 (0.01-2.27) | 0.63 (0.37-0.89) |
| Greenland | 0.16 (0-0.65) | 0.19 (0-0.69) | 0.19 | 0.46 (0-1.93) | 0.27 (0-0.98) | -1.77 (-1.87--1.67) |
| Grenada | 0.45 (0-1.84) | 0.55 (0-2.32) | 0.22 | 0.6 (0-2.46) | 0.49 (0-2.06) | -1.7 (-2.45--0.95) |
| Guam | 1.19 (0.11-3.37) | 1.03 (0.07-3.13) | -0.13 | 1.9 (0.17-5.46) | 0.48 (0.03-1.5) | -4.56 (-4.75--4.37) |
| Guatemala | 5.12 (0.26-16.79) | 11.16 (0.62-33.31) | 1.18 | 0.15 (0.01-0.49) | 0.1 (0.01-0.3) | -1.98 (-2.24--1.72) |
| Guinea | 4.61 (0-23.68) | 9.36 (0-51.11) | 1.03 | 0.14 (0-0.73) | 0.17 (0-0.97) | 0.44 (0.32-0.55) |
| Guinea-Bissau | 0.81 (0-4.02) | 1.39 (0-6.92) | 0.72 | 0.21 (0-1.07) | 0.19 (0-0.96) | -0.4 (-0.48--0.32) |
| Guyana | 0.83 (0-3.42) | 2.43 (0-10.22) | 1.93 | 0.23 (0-0.96) | 0.39 (0-1.62) | 1.02 (0.27-1.77) |
| Haiti | 7.52 (0.01-31.26) | 14.45 (0.01-58.83) | 0.92 | 0.27 (0-1.09) | 0.23 (0-0.91) | -0.69 (-0.76--0.62) |
| Honduras | 3.59 (0.14-11.24) | 15.85 (0.66-49.76) | 3.42 | 0.18 (0.01-0.55) | 0.25 (0.01-0.8) | 1.34 (1.23-1.46) |
| Hungary | 166.35 (37.55-343.18) | 188.73 (36.73-425.87) | 0.13 | 1.19 (0.27-2.48) | 1.07 (0.21-2.47) | -0.91 (-1.15--0.67) |
| Iceland | 1.66 (0.01-6.14) | 1.81 (0.02-6.64) | 0.09 | 0.58 (0-2.13) | 0.31 (0-1.13) | -2.4 (-2.7--2.1) |
| India | 534.79 (12.87-1956.99) | 2387.87 (91.71-8146.28) | 3.47 | 0.11 (0-0.41) | 0.19 (0.01-0.67) | 2.01 (1.85-2.17) |
| Indonesia | 175.63 (28.14-432.91) | 438.57 (49.91-1209.23) | 1.5 | 0.18 (0.03-0.43) | 0.18 (0.02-0.51) | -0.31 (-0.45--0.18) |
| Iran (Islamic Republic of) | 4.67 (0-24.41) | 24.47 (0.01-130.42) | 4.24 | 0.02 (0-0.09) | 0.03 (0-0.16) | 2.81 (2.41-3.22) |
| Iraq | 1.64 (0-8.67) | 5.84 (0-30.98) | 2.56 | 0.02 (0-0.1) | 0.02 (0-0.11) | 0.22 (0.15-0.29) |
| Ireland | 18.52 (0.01-82.77) | 14.77 (0.01-68.46) | -0.2 | 0.45 (0-1.99) | 0.19 (0-0.85) | -2.94 (-3.22--2.65) |
| Israel | 10.9 (0.04-43.36) | 15.54 (0.08-62.31) | 0.43 | 0.23 (0-0.9) | 0.13 (0-0.5) | -2.13 (-2.32--1.94) |
| Italy | 392.82 (26.79-1184.24) | 482.64 (19.22-1668.91) | 0.23 | 0.47 (0.03-1.41) | 0.38 (0.02-1.23) | -1.16 (-1.45--0.87) |
| Jamaica | 3.93 (0.01-16) | 6.01 (0.01-25.19) | 0.53 | 0.21 (0-0.87) | 0.19 (0-0.82) | -0.62 (-1--0.23) |
| Japan | 1377.02 (256.2-3157.23) | 3631.82 (222.95-10816.7) | 1.64 | 0.81 (0.15-1.86) | 1.05 (0.07-3.12) | 0.72 (0.57-0.87) |
| Jordan | 1.11 (0-5.93) | 5.29 (0-29.47) | 3.77 | 0.07 (0-0.39) | 0.06 (0-0.34) | -0.67 (-0.86--0.48) |
| Kazakhstan | 50.33 (3.98-149.52) | 65.52 (2.13-229.75) | 0.3 | 0.37 (0.03-1.09) | 0.34 (0.01-1.19) | -1.32 (-1.74--0.9) |
| Kenya | 11.6 (0.56-42.9) | 27.1 (0.75-111.95) | 1.34 | 0.15 (0.01-0.55) | 0.13 (0-0.5) | -0.54 (-0.72--0.36) |
| Kiribati | 0.04 (0-0.11) | 0.08 (0.01-0.25) | 1 | 0.11 (0.01-0.33) | 0.12 (0.01-0.36) | -0.21 (-0.36--0.05) |
| Kuwait | 1.18 (0.01-4.6) | 4.16 (0.03-16.73) | 2.53 | 0.13 (0-0.54) | 0.11 (0-0.44) | 0 (-1.12-1.12) |
| Kyrgyzstan | 2.49 (0.2-7.54) | 8.57 (0.25-29.85) | 2.44 | 0.08 (0.01-0.25) | 0.17 (0.01-0.57) | 2.66 (2.05-3.27) |
| Lao People's Democratic Republic | 6.17 (0.93-15.68) | 11.22 (0.95-32.39) | 0.82 | 0.3 (0.04-0.78) | 0.26 (0.02-0.75) | -0.7 (-0.79--0.61) |
| Latvia | 10.93 (0.16-39.6) | 14.85 (0.19-53.01) | 0.36 | 0.31 (0-1.12) | 0.43 (0.01-1.57) | 0.29 (-0.08-0.66) |
| Lebanon | 4.14 (0-25.83) | 6.95 (0-37.09) | 0.68 | 0.18 (0-1.13) | 0.11 (0-0.61) | -1.29 (-1.59--1) |
| Lesotho | 0.96 (0-4.91) | 1.43 (0-7.5) | 0.49 | 0.12 (0-0.6) | 0.13 (0-0.69) | 0.6 (0.4-0.81) |
| Liberia | 1.94 (0-10.65) | 3.2 (0-17.1) | 0.65 | 0.17 (0-0.96) | 0.16 (0-0.86) | -0.33 (-0.48--0.17) |
| Libya | 0.17 (0-0.9) | 1.32 (0-6.86) | 6.76 | 0.01 (0-0.04) | 0.02 (0-0.11) | 3.92 (3.56-4.27) |
| Lithuania | 9.79 (0-40.96) | 15.82 (0-67.83) | 0.62 | 0.22 (0-0.9) | 0.31 (0-1.29) | 0.75 (0.5-1) |
| Luxembourg | 2.37 (0.01-9.46) | 2.25 (0.01-8.95) | -0.05 | 0.44 (0-1.77) | 0.21 (0-0.84) | -2.94 (-3.17--2.71) |
| Madagascar | 39.31 (1.78-135.2) | 41.66 (0.64-164.61) | 0.06 | 0.8 (0.04-2.73) | 0.41 (0.01-1.53) | -2.55 (-2.79--2.32) |
| Malawi | 12.29 (0.42-43.67) | 18.84 (0.28-78.37) | 0.53 | 0.34 (0.01-1.22) | 0.27 (0-1.07) | -1.19 (-1.4--0.98) |
| Malaysia | 102.53 (17.71-245.65) | 248.37 (30.58-639.13) | 1.42 | 1.17 (0.2-2.76) | 0.9 (0.1-2.32) | -1.84 (-2.27--1.4) |
| Maldives | 0.21 (0.03-0.54) | 0.48 (0.03-1.79) | 1.29 | 0.25 (0.03-0.65) | 0.14 (0.01-0.49) | -2.4 (-2.6--2.2) |
| Mali | 3.9 (0-19.56) | 8.82 (0-49.35) | 1.26 | 0.1 (0-0.5) | 0.1 (0-0.57) | 0.08 (-0.1-0.27) |
| Malta | 1.41 (0.02-4.93) | 1.85 (0.05-6.49) | 0.31 | 0.33 (0.01-1.15) | 0.21 (0.01-0.7) | -1.89 (-2.21--1.58) |
| Marshall Islands | 0.11 (0.01-0.34) | 0.22 (0.01-0.67) | 1 | 0.8 (0.06-2.41) | 0.73 (0.04-2.18) | -0.59 (-0.69--0.49) |
| Mauritania | 2.18 (0-10.69) | 4.05 (0-21.85) | 0.86 | 0.22 (0-1.1) | 0.19 (0-1.05) | -0.79 (-0.95--0.63) |
| Mauritius | 4.06 (0.67-9.86) | 4.58 (0.57-12.24) | 0.13 | 0.57 (0.09-1.38) | 0.25 (0.03-0.67) | -2.73 (-3.27--2.19) |
| Mexico | 46.57 (0.92-168.73) | 111.87 (1.85-403.67) | 1.4 | 0.11 (0-0.4) | 0.09 (0-0.32) | -1.24 (-1.43--1.04) |
| Micronesia (Federated States of) | 0.43 (0.04-1.36) | 0.54 (0.03-1.58) | 0.26 | 1 (0.08-3.16) | 0.83 (0.05-2.46) | -0.95 (-1.06--0.84) |
| Monaco | 0.34 (0-1.47) | 0.45 (0-1.91) | 0.32 | 0.51 (0-2.17) | 0.49 (0-2) | 0.03 (-0.1-0.17) |
| Mongolia | 0.81 (0.06-2.46) | 2.81 (0.09-9.72) | 2.47 | 0.08 (0.01-0.23) | 0.11 (0-0.39) | 1.25 (0.82-1.68) |
| Montenegro | 18.81 (3.59-43.35) | 27.92 (5-67.1) | 0.48 | 2.96 (0.55-6.81) | 2.9 (0.52-7.02) | 0.03 (-0.12-0.19) |
| Morocco | 1.19 (0-6.61) | 7.82 (0-41.83) | 5.57 | 0.01 (0-0.04) | 0.02 (0-0.12) | 4.11 (3.87-4.36) |
| Mozambique | 22.88 (0.95-79.35) | 35.85 (0.45-148.46) | 0.57 | 0.42 (0.02-1.43) | 0.35 (0-1.37) | -0.58 (-0.66--0.5) |
| Myanmar | 64.51 (8.74-156.18) | 119.42 (13.05-316.2) | 0.85 | 0.28 (0.04-0.69) | 0.25 (0.03-0.67) | -0.59 (-0.7--0.48) |
| Namibia | 1.2 (0-5.77) | 2.07 (0-9.99) | 0.72 | 0.18 (0-0.9) | 0.14 (0-0.72) | -1.07 (-1.26--0.88) |
| Nauru | 0.05 (0-0.13) | 0.05 (0-0.15) | 0 | 1.17 (0.12-3.39) | 1.11 (0.06-3.36) | -0.38 (-0.48--0.28) |
| Nepal | 6.07 (0-28.92) | 40.84 (0.28-156.45) | 5.73 | 0.07 (0-0.34) | 0.19 (0-0.72) | 3.82 (3.55-4.1) |
| Netherlands | 114.4 (0.18-546.89) | 103.76 (0.25-447.14) | -0.09 | 0.57 (0-2.73) | 0.3 (0-1.26) | -2.47 (-2.81--2.12) |
| New Zealand | 34.99 (0.09-142.95) | 32.37 (0.05-138.9) | -0.07 | 0.88 (0-3.58) | 0.38 (0-1.63) | -3.22 (-3.43--3) |
| Nicaragua | 1.28 (0.07-3.93) | 3.98 (0.22-12.09) | 2.11 | 0.08 (0-0.25) | 0.08 (0-0.25) | -0.1 (-0.31-0.11) |
| Niger | 2.61 (0-13.18) | 7.31 (0-39.87) | 1.8 | 0.1 (0-0.53) | 0.09 (0-0.5) | -0.38 (-0.47--0.29) |
| Nigeria | 76.48 (0-440.73) | 114.89 (0.03-640.88) | 0.5 | 0.18 (0-1.06) | 0.13 (0-0.71) | -1.81 (-2.09--1.53) |
| Niue | 0.02 (0-0.05) | 0.01 (0-0.04) | -0.5 | 0.78 (0.07-2.32) | 0.63 (0.05-1.85) | -1.08 (-1.21--0.94) |
| North Macedonia | 15.08 (3.14-34.22) | 29 (4.19-75.48) | 0.92 | 0.81 (0.17-1.81) | 0.87 (0.13-2.28) | -0.05 (-0.18-0.09) |
| Northern Mariana Islands | 0.15 (0.01-0.51) | 0.35 (0.02-1.17) | 1.33 | 1.33 (0.11-3.94) | 0.74 (0.05-2.35) | -2.89 (-3.21--2.57) |
| Norway | 38.85 (0.4-156.74) | 34.83 (0.13-137.32) | -0.1 | 0.59 (0.01-2.31) | 0.34 (0-1.34) | -2.08 (-2.46--1.69) |
| Oman | 0.11 (0-0.59) | 0.98 (0-6.19) | 7.91 | 0.01 (0-0.07) | 0.04 (0-0.21) | 3.93 (3.5-4.36) |
| Pakistan | 45.89 (0.06-201.09) | 234.74 (3.16-898.36) | 4.12 | 0.08 (0-0.37) | 0.2 (0-0.75) | 3.11 (2.74-3.49) |
| Palau | 0.07 (0.01-0.22) | 0.13 (0.01-0.43) | 0.86 | 0.84 (0.06-2.51) | 0.65 (0.04-2.11) | -1.11 (-1.2--1.03) |
| Palestine | 0.33 (0-1.95) | 1.25 (0-7.06) | 2.79 | 0.04 (0-0.22) | 0.04 (0-0.26) | 0.71 (0.63-0.79) |
| Panama | 7.89 (0.44-23.48) | 17.59 (0.9-54.53) | 1.23 | 0.53 (0.03-1.57) | 0.4 (0.02-1.24) | -1.39 (-1.6--1.19) |
| Papua New Guinea | 6.24 (0.33-18.9) | 18.17 (0.92-59.98) | 1.91 | 0.42 (0.02-1.23) | 0.42 (0.03-1.33) | -0.29 (-0.4--0.17) |
| Paraguay | 10.06 (0.22-33.89) | 34.71 (0.71-118.49) | 2.45 | 0.45 (0.01-1.5) | 0.59 (0.01-2.01) | 0.83 (0.72-0.94) |
| Peru | 19.73 (0.4-67.01) | 42.13 (0.96-146.34) | 1.14 | 0.17 (0-0.56) | 0.13 (0-0.44) | -1.24 (-1.43--1.05) |
| Philippines | 120.9 (19.56-302.83) | 327.48 (38.23-921.75) | 1.71 | 0.42 (0.07-1.08) | 0.41 (0.05-1.14) | -0.36 (-0.46--0.25) |
| Poland | 497.79 (49.6-1374.81) | 682.89 (72.64-1906.92) | 0.37 | 1.14 (0.11-3.14) | 0.97 (0.11-2.73) | -0.69 (-0.89--0.49) |
| Portugal | 21.76 (0.12-90.11) | 48.45 (0.96-176.24) | 1.23 | 0.16 (0-0.66) | 0.23 (0.01-0.82) | 1.1 (0.89-1.32) |
| Puerto Rico | 9.8 (0.04-38.34) | 7.22 (0.01-30.16) | -0.26 | 0.27 (0-1.06) | 0.1 (0-0.4) | -4.14 (-4.42--3.86) |
| Qatar | 0.12 (0-0.58) | 0.76 (0-4.61) | 5.33 | 0.07 (0-0.41) | 0.05 (0-0.32) | -1.61 (-2--1.22) |
| Republic of Korea | 168.05 (16.54-465.58) | 603.87 (73.27-1624.59) | 2.59 | 0.6 (0.06-1.65) | 0.65 (0.08-1.73) | 0.43 (0.16-0.7) |
| Republic of Moldova | 4.2 (0.01-17.97) | 7.68 (0.01-33.69) | 0.83 | 0.09 (0-0.4) | 0.13 (0-0.58) | 0.92 (0.65-1.18) |
| Romania | 151.99 (30.38-331.16) | 227.45 (38.3-550.81) | 0.5 | 0.56 (0.11-1.2) | 0.68 (0.12-1.61) | 0.37 (0.14-0.61) |
| Russian Federation | 1078.77 (72.9-3284.88) | 2052.29 (131.16-6202.15) | 0.9 | 0.59 (0.04-1.8) | 0.9 (0.06-2.73) | 1.02 (0.67-1.37) |
| Rwanda | 19.3 (0.56-63.87) | 18.33 (0.3-70.5) | -0.05 | 0.72 (0.02-2.4) | 0.32 (0.01-1.24) | -3.53 (-3.96--3.1) |
| Saint Kitts and Nevis | 0.17 (0-0.67) | 0.16 (0-0.65) | -0.06 | 0.43 (0-1.69) | 0.25 (0-1.04) | -2.2 (-2.43--1.97) |
| Saint Lucia | 0.78 (0-3.12) | 1 (0-4.22) | 0.28 | 0.96 (0-3.86) | 0.43 (0-1.79) | -3.72 (-4.07--3.35) |
| Saint Vincent and the Grenadines | 0.23 (0-0.93) | 0.32 (0-1.3) | 0.39 | 0.33 (0-1.29) | 0.23 (0-0.93) | -1.75 (-2.05--1.45) |
| Samoa | 0.14 (0-0.79) | 0.27 (0-1.3) | 0.93 | 0.21 (0-1.07) | 0.23 (0-1.05) | -0.07 (-0.24-0.11) |
| San Marino | 0.11 (0-0.45) | 0.14 (0-0.6) | 0.27 | 0.32 (0-1.24) | 0.19 (0-0.84) | -0.53 (-0.91--0.15) |
| Sao Tome and Principe | 0.08 (0-0.45) | 0.18 (0-0.96) | 1.25 | 0.12 (0-0.7) | 0.17 (0-0.86) | 0.95 (0.9-1.01) |
| Saudi Arabia | 0.28 (0-1.57) | 3.21 (0-18.43) | 10.46 | 0 (0-0.02) | 0.01 (0-0.06) | 4.18 (3.69-4.67) |
| Senegal | 4.85 (0-25.21) | 12.27 (0-67.25) | 1.53 | 0.15 (0-0.79) | 0.16 (0-0.85) | -0.06 (-0.22-0.11) |
| Serbia | 147.17 (30.52-317.15) | 213.83 (39.39-496.23) | 0.45 | 1.32 (0.27-2.86) | 1.34 (0.24-3.11) | 0.11 (-0.02-0.25) |
| Seychelles | 0.18 (0.03-0.45) | 0.26 (0.02-0.81) | 0.44 | 0.32 (0.04-0.8) | 0.23 (0.02-0.72) | -1.01 (-1.17--0.86) |
| Sierra Leone | 2.55 (0-13.91) | 4.78 (0-26.76) | 0.87 | 0.12 (0-0.67) | 0.12 (0-0.67) | 0.07 (-0.01-0.16) |
| Singapore | 21.29 (2.64-54.67) | 56.92 (4.47-153.79) | 1.67 | 0.96 (0.12-2.49) | 0.66 (0.05-1.79) | -1.04 (-1.27--0.8) |
| Slovakia | 64.42 (14.17-144.13) | 79.09 (13.49-183.51) | 0.23 | 1.09 (0.24-2.44) | 0.86 (0.15-2) | -0.8 (-0.88--0.72) |
| Slovenia | 30.14 (5.94-65.1) | 30.31 (5.45-70.72) | 0.01 | 1.23 (0.24-2.64) | 0.7 (0.13-1.65) | -2.21 (-2.47--1.95) |
| Solomon Islands | 0.51 (0.04-1.59) | 1.35 (0.09-4.19) | 1.65 | 0.48 (0.04-1.44) | 0.48 (0.03-1.43) | -0.31 (-0.45--0.17) |
| Somalia | 7.04 (0.24-26.08) | 8.79 (0.11-36.49) | 0.25 | 0.33 (0.01-1.16) | 0.16 (0-0.61) | -2.72 (-2.93--2.52) |
| South Africa | 44.81 (0.08-204.61) | 53.77 (0.01-288.97) | 0.2 | 0.2 (0-0.91) | 0.11 (0-0.6) | -2.53 (-2.83--2.24) |
| South Sudan | 13 (0.5-48.82) | 7.85 (0.09-31.78) | -0.4 | 0.52 (0.02-1.92) | 0.23 (0-0.91) | -3.2 (-3.5--2.89) |
| Spain | 84.61 (0.12-406.04) | 145.82 (1.79-636.68) | 0.72 | 0.17 (0-0.78) | 0.18 (0-0.73) | -0.2 (-0.48-0.08) |
| Sri Lanka | 12.64 (1.76-31.29) | 24.29 (2.37-64.45) | 0.92 | 0.12 (0.02-0.3) | 0.09 (0.01-0.24) | -0.97 (-1.13--0.82) |
| Sudan | 0.72 (0-4.46) | 4.57 (0-24.19) | 5.35 | 0.01 (0-0.04) | 0.02 (0-0.1) | 3.87 (3.65-4.09) |
| Suriname | 0.79 (0-3.29) | 1.61 (0-6.83) | 1.04 | 0.33 (0-1.35) | 0.26 (0-1.08) | -0.93 (-1.12--0.74) |
| Sweden | 110.45 (0.82-413.73) | 81.28 (0.2-328.68) | -0.26 | 0.76 (0.01-2.82) | 0.38 (0-1.51) | -2.52 (-2.87--2.17) |
| Switzerland | 59.92 (0.17-241.49) | 44.31 (0.23-180.41) | -0.26 | 0.59 (0-2.34) | 0.25 (0-1) | -2.86 (-3.04--2.68) |
| Syrian Arab Republic | 1.23 (0-7.11) | 3.47 (0-19.95) | 1.82 | 0.02 (0-0.12) | 0.02 (0-0.14) | 0.35 (0.24-0.46) |
| Taiwan | 56.46 (2.49-168.89) | 208.66 (7.96-697.37) | 2.7 | 0.35 (0.02-1.04) | 0.5 (0.02-1.68) | 0.02 (-0.67-0.72) |
| Tajikistan | 2.27 (0.21-6.53) | 2.93 (0.1-10.05) | 0.29 | 0.08 (0.01-0.23) | 0.05 (0-0.17) | -2 (-2.21--1.8) |
| Thailand | 225.56 (30.7-596.31) | 618.6 (65.06-1738.63) | 1.74 | 0.69 (0.09-1.8) | 0.57 (0.06-1.61) | -1.25 (-1.45--1.05) |
| Timor-Leste | 0.48 (0.07-1.21) | 1.48 (0.14-4.17) | 2.08 | 0.19 (0.03-0.47) | 0.18 (0.02-0.5) | -0.32 (-0.47--0.17) |
| Togo | 2.11 (0-10.65) | 7.11 (0-37.72) | 2.37 | 0.17 (0-0.86) | 0.18 (0-0.96) | -0.13 (-0.24--0.02) |
| Tokelau | 0.01 (0-0.03) | 0.01 (0-0.03) | 0 | 0.88 (0.07-2.62) | 0.73 (0.05-2.22) | -1.06 (-1.27--0.86) |
| Tonga | 0.38 (0.04-1.1) | 0.57 (0.04-1.81) | 0.5 | 0.76 (0.07-2.13) | 0.73 (0.06-2.32) | -0.48 (-0.72--0.23) |
| Trinidad and Tobago | 4.37 (0.01-17.74) | 7.15 (0.01-29.08) | 0.64 | 0.53 (0-2.17) | 0.37 (0-1.49) | -1.72 (-2.01--1.42) |
| Tunisia | 0.53 (0-3.09) | 3.38 (0-19.66) | 5.38 | 0.01 (0-0.06) | 0.02 (0-0.14) | 3.47 (3.17-3.76) |
| T眉rkiye | 35.52 (0-232.93) | 93.97 (0-595.37) | 1.65 | 0.09 (0-0.61) | 0.1 (0-0.61) | 0.12 (-0.19-0.43) |
| Turkmenistan | 5.53 (0.47-16.77) | 14.95 (0.39-51.97) | 1.7 | 0.28 (0.03-0.85) | 0.34 (0.01-1.19) | 0.37 (0.16-0.58) |
| Tuvalu | 0.05 (0-0.14) | 0.07 (0.01-0.2) | 0.4 | 0.78 (0.06-2.35) | 0.68 (0.05-2.02) | -0.77 (-0.89--0.66) |
| Uganda | 20.63 (0.77-75.47) | 31.81 (0.45-126.58) | 0.54 | 0.34 (0.01-1.18) | 0.24 (0-0.9) | -1.52 (-1.76--1.28) |
| Ukraine | 171.88 (0.32-700.26) | 207.39 (0.37-954.78) | 0.21 | 0.24 (0-0.99) | 0.3 (0-1.36) | 0.02 (-0.43-0.46) |
| United Arab Emirates | 0.57 (0-2.99) | 3.82 (0-21.4) | 5.7 | 0.07 (0-0.42) | 0.07 (0-0.4) | 1.26 (0.62-1.9) |
| United Kingdom | 509.48 (2.1-2173.87) | 431.18 (2.83-1855.92) | -0.15 | 0.54 (0-2.3) | 0.31 (0-1.33) | -1.8 (-2.22--1.38) |
| United Republic of Tanzania | 65.83 (3.92-197.9) | 104.67 (4.26-380.05) | 0.59 | 0.64 (0.04-1.93) | 0.43 (0.02-1.5) | -2.06 (-2.28--1.84) |
| United States of America | 1286.69 (0.93-5836.21) | 1830.45 (34.7-6635.46) | 0.42 | 0.4 (0-1.82) | 0.35 (0.01-1.23) | -0.77 (-1.18--0.36) |
| United States Virgin Islands | 0.29 (0-1.2) | 0.37 (0-1.51) | 0.28 | 0.38 (0-1.52) | 0.21 (0-0.83) | -2.25 (-2.49--2.01) |
| Uruguay | 51.93 (1.29-163.44) | 47.92 (1.14-162.23) | -0.08 | 1.33 (0.03-4.19) | 0.9 (0.02-3.08) | -1.72 (-1.96--1.47) |
| Uzbekistan | 7.06 (0.55-22.5) | 45.11 (1.54-161.9) | 5.39 | 0.06 (0-0.19) | 0.18 (0.01-0.64) | 3.94 (3.06-4.82) |
| Vanuatu | 0.23 (0.02-0.68) | 0.63 (0.05-2.04) | 1.74 | 0.42 (0.03-1.19) | 0.39 (0.03-1.16) | -0.58 (-0.69--0.46) |
| Venezuela (Bolivarian Republic of) | 46.2 (2.4-140.53) | 115.06 (6.1-348.67) | 1.49 | 0.47 (0.02-1.38) | 0.38 (0.02-1.16) | -1.27 (-1.58--0.96) |
| Viet Nam | 90.89 (12.47-227.79) | 248.6 (23.45-695.17) | 1.74 | 0.23 (0.03-0.57) | 0.26 (0.02-0.71) | 0.06 (-0.07-0.19) |
| Yemen | 0.39 (0-2.16) | 3.29 (0-17.2) | 7.44 | 0.01 (0-0.04) | 0.02 (0-0.1) | 3.99 (3.62-4.36) |
| Zambia | 14.64 (0.61-46.61) | 36.93 (0.49-162.25) | 1.52 | 0.57 (0.02-1.86) | 0.6 (0.01-2.54) | -0.03 (-0.42-0.37) |
| Zimbabwe | 11.49 (0.02-47.9) | 25.81 (0.03-101.15) | 1.25 | 0.29 (0-1.23) | 0.35 (0-1.44) | 0.43 (0.25-0.61) |

Supplementary table 1. The DALYs of aortic aneurysm attributable to diet high in sodium cases and rates in 1990 and 2021 across 204 countries, and the trends from 1990 to 2021.

| Location | 1990_Death cases (95% UI) | 2021_Death cases (95% UI) | Percentage change | 1990_ASMR_per 100000(95% UI) | 2021_ASMR_per 100 000(95% UI) | EAPC (95% CI) |
| --- | --- | --- | --- | --- | --- | --- |
| Afghanistan | 0.01 (0-0.09) | 0.06 (0-0.36) | 5 | 0 (0-0) | 0 (0-0) | 4.24 (3.96-4.51) |
| Albania | 0.33 (0.07-0.74) | 0.75 (0.13-1.91) | 1.27 | 0.02 (0-0.04) | 0.02 (0-0.04) | 0.13 (0-0.26) |
| Algeria | 0.04 (0-0.23) | 0.29 (0-1.63) | 6.25 | 0 (0-0) | 0 (0-0.01) | 3.73 (3.39-4.08) |
| American Samoa | 0 (0-0.01) | 0.01 (0-0.02) | Inf | 0.02 (0-0.09) | 0.02 (0-0.07) | -1.24 (-1.47--1.01) |
| Andorra | 0.01 (0-0.07) | 0.02 (0-0.12) | 1 | 0.03 (0-0.13) | 0.01 (0-0.08) | -1.58 (-1.83--1.34) |
| Angola | 0.21 (0-1.18) | 0.86 (0-4.64) | 3.1 | 0.01 (0-0.04) | 0.01 (0-0.05) | 0.75 (0.62-0.87) |
| Antigua and Barbuda | 0.01 (0-0.05) | 0.01 (0-0.05) | 0 | 0.02 (0-0.08) | 0.01 (0-0.05) | -2.48 (-2.79--2.17) |
| Argentina | 16.64 (0.24-56.37) | 14.64 (0.25-50.35) | -0.12 | 0.05 (0-0.18) | 0.03 (0-0.09) | -2.58 (-2.79--2.37) |
| Armenia | 1.37 (0.1-3.87) | 3.23 (0.13-11) | 1.36 | 0.05 (0-0.15) | 0.08 (0-0.26) | 1.25 (0.82-1.69) |
| Australia | 3.59 (0-19.64) | 3.23 (0-18.22) | -0.1 | 0.02 (0-0.1) | 0.01 (0-0.04) | -3.53 (-3.7--3.36) |
| Austria | 3.16 (0.05-11) | 3.08 (0.07-9.78) | -0.03 | 0.03 (0-0.09) | 0.02 (0-0.05) | -1.81 (-1.99--1.64) |
| Azerbaijan | 0.42 (0.03-1.26) | 1.04 (0.03-3.91) | 1.48 | 0.01 (0-0.03) | 0.01 (0-0.04) | 1.23 (0.9-1.57) |
| Bahamas | 0.03 (0-0.13) | 0.06 (0-0.24) | 1 | 0.02 (0-0.09) | 0.02 (0-0.07) | -1.65 (-2.02--1.29) |
| Bahrain | 0 (0-0.01) | 0.01 (0-0.08) | Inf | 0 (0-0.01) | 0 (0-0.01) | 0.01 (-0.34-0.37) |
| Bangladesh | 1.85 (0-8.31) | 11.13 (0.07-43.85) | 5.02 | 0 (0-0.02) | 0.01 (0-0.04) | 2.68 (2.32-3.04) |
| Barbados | 0.04 (0-0.18) | 0.04 (0-0.21) | 0 | 0.01 (0-0.06) | 0.01 (0-0.04) | -2.26 (-2.62--1.91) |
| Belarus | 1.35 (0-5.98) | 2.14 (0-9.76) | 0.59 | 0.01 (0-0.05) | 0.01 (0-0.06) | 0.33 (-0.1-0.75) |
| Belgium | 8.34 (0.23-27.27) | 4.57 (0.07-15.54) | -0.45 | 0.05 (0-0.17) | 0.02 (0-0.06) | -3.91 (-4.08--3.74) |
| Belize | 0.01 (0-0.02) | 0.01 (0-0.05) | 0 | 0.01 (0-0.03) | 0 (0-0.02) | -1.67 (-2.44--0.9) |
| Benin | 0.14 (0-0.67) | 0.34 (0-1.66) | 1.43 | 0.01 (0-0.04) | 0.01 (0-0.04) | -0.17 (-0.25--0.1) |
| Bermuda | 0.05 (0-0.18) | 0.04 (0-0.16) | -0.2 | 0.08 (0-0.31) | 0.03 (0-0.1) | -3.85 (-4.01--3.7) |
| Bhutan | 0.01 (0-0.03) | 0.06 (0-0.25) | 5 | 0 (0-0.02) | 0.01 (0-0.05) | 3.91 (3.67-4.14) |
| Bolivia (Plurinational State of) | 0.37 (0.01-1.23) | 1.07 (0.02-3.83) | 1.89 | 0.01 (0-0.04) | 0.01 (0-0.05) | -0.07 (-0.15-0.01) |
| Bosnia and Herzegovina | 1.7 (0.29-3.72) | 3.37 (0.53-8.08) | 0.98 | 0.04 (0.01-0.1) | 0.05 (0.01-0.13) | 0.64 (0.51-0.77) |
| Botswana | 0.04 (0-0.19) | 0.07 (0-0.39) | 0.75 | 0.01 (0-0.04) | 0.01 (0-0.03) | -1.64 (-1.8--1.48) |
| Brazil | 26.26 (1.24-87.11) | 82.83 (2.5-268.85) | 2.15 | 0.03 (0-0.1) | 0.03 (0-0.11) | -0.12 (-0.37-0.12) |
| Brunei Darussalam | 0.07 (0.01-0.19) | 0.19 (0.02-0.52) | 1.71 | 0.08 (0.01-0.22) | 0.07 (0.01-0.19) | -0.23 (-0.38--0.07) |
| Bulgaria | 4.78 (0.98-10.68) | 6.52 (1.24-15.96) | 0.36 | 0.04 (0.01-0.1) | 0.05 (0.01-0.11) | 0.14 (-0.06-0.35) |
| Burkina Faso | 0.25 (0-1.37) | 0.71 (0-3.44) | 1.84 | 0.01 (0-0.04) | 0.01 (0-0.05) | 1.16 (1.08-1.24) |
| Burundi | 0.6 (0.02-2.01) | 0.43 (0.01-1.86) | -0.28 | 0.03 (0-0.1) | 0.01 (0-0.05) | -3.66 (-4.06--3.25) |
| Cabo Verde | 0.02 (0-0.1) | 0.04 (0-0.19) | 1 | 0.01 (0-0.04) | 0.01 (0-0.05) | 0.51 (0.23-0.78) |
| Cambodia | 0.42 (0.06-1.11) | 1.24 (0.1-3.74) | 1.95 | 0.01 (0-0.03) | 0.01 (0-0.04) | 0.26 (0.11-0.41) |
| Cameroon | 0.39 (0-1.89) | 1 (0-4.45) | 1.56 | 0.01 (0-0.05) | 0.01 (0-0.04) | -0.89 (-1.18--0.61) |
| Canada | 14.23 (0.08-52.76) | 12.36 (0.11-48.58) | -0.13 | 0.04 (0-0.16) | 0.02 (0-0.06) | -3.74 (-4.01--3.48) |
| Central African Republic | 0.06 (0-0.35) | 0.11 (0-0.61) | 0.83 | 0.01 (0-0.04) | 0.01 (0-0.04) | -0.27 (-0.44--0.1) |
| Chad | 0.15 (0-0.76) | 0.32 (0-1.82) | 1.13 | 0.01 (0-0.03) | 0.01 (0-0.04) | 0.42 (0.31-0.53) |
| Chile | 2.05 (0.05-6.46) | 4.13 (0.09-13.99) | 1.01 | 0.02 (0-0.07) | 0.02 (0-0.05) | -0.93 (-1.11--0.75) |
| China | 65.34 (12.85-152.2) | 206.85 (37.18-508.9) | 2.17 | 0.01 (0-0.02) | 0.01 (0-0.02) | 1.07 (0.89-1.26) |
| Colombia | 9.93 (1.41-25.27) | 23.15 (3.55-56.72) | 1.33 | 0.06 (0.01-0.15) | 0.04 (0.01-0.1) | -2.53 (-2.97--2.08) |
| Comoros | 0.05 (0-0.16) | 0.07 (0-0.28) | 0.4 | 0.03 (0-0.1) | 0.02 (0-0.07) | -2.31 (-2.54--2.08) |
| Congo | 0.09 (0-0.48) | 0.22 (0-1.15) | 1.44 | 0.01 (0-0.06) | 0.01 (0-0.05) | -0.24 (-0.43--0.05) |
| Cook Islands | 0.01 (0-0.02) | 0.01 (0-0.03) | 0 | 0.05 (0.01-0.15) | 0.04 (0-0.12) | -1.41 (-1.53--1.3) |
| Costa Rica | 0.37 (0.02-1.11) | 1.27 (0.08-3.66) | 2.43 | 0.02 (0-0.07) | 0.02 (0-0.07) | -0.29 (-0.51--0.06) |
| C么te d'Ivoire | 0.35 (0-1.6) | 0.95 (0-4.67) | 1.71 | 0.01 (0-0.05) | 0.01 (0-0.05) | -0.8 (-0.98--0.62) |
| Croatia | 2.91 (0.56-6.36) | 4.96 (0.89-11.74) | 0.7 | 0.05 (0.01-0.11) | 0.05 (0.01-0.12) | -0.04 (-0.24-0.17) |
| Cuba | 4.25 (0.03-16.3) | 5.1 (0.01-20.28) | 0.2 | 0.04 (0-0.17) | 0.02 (0-0.1) | -2.38 (-2.61--2.15) |
| Cyprus | 0.25 (0-1.07) | 0.42 (0-1.75) | 0.68 | 0.04 (0-0.16) | 0.02 (0-0.08) | -2.39 (-2.58--2.2) |
| Czechia | 9.09 (1.76-20.43) | 12.39 (2.05-28.63) | 0.36 | 0.07 (0.01-0.15) | 0.05 (0.01-0.13) | -0.83 (-1.22--0.44) |
| Democratic People's Republic of Korea | 2.05 (0.35-4.87) | 3.21 (0.5-8.41) | 0.57 | 0.01 (0-0.03) | 0.01 (0-0.03) | -0.66 (-0.77--0.56) |
| Democratic Republic of the Congo | 0.51 (0-3.49) | 1.12 (0-8.11) | 1.2 | 0 (0-0.03) | 0 (0-0.03) | -0.26 (-0.68-0.15) |
| Denmark | 2.51 (0.01-9.94) | 2.79 (0.01-11.88) | 0.11 | 0.03 (0-0.12) | 0.02 (0-0.09) | -1.44 (-1.74--1.15) |
| Djibouti | 0.03 (0-0.09) | 0.07 (0-0.26) | 1.33 | 0.03 (0-0.09) | 0.01 (0-0.05) | -2.49 (-2.69--2.28) |
| Dominica | 0.01 (0-0.06) | 0.02 (0-0.07) | 1 | 0.03 (0-0.1) | 0.02 (0-0.09) | -0.94 (-1.17--0.71) |
| Dominican Republic | 0.31 (0-1.16) | 0.96 (0-3.84) | 2.1 | 0.01 (0-0.04) | 0.01 (0-0.04) | -0.39 (-0.61--0.16) |
| Ecuador | 0.74 (0.02-2.45) | 2.07 (0.03-7.24) | 1.8 | 0.02 (0-0.05) | 0.01 (0-0.05) | -0.14 (-0.48-0.21) |
| Egypt | 0.23 (0-1.26) | 0.65 (0-3.57) | 1.83 | 0 (0-0.01) | 0 (0-0.01) | 0.56 (0.46-0.65) |
| El Salvador | 0.17 (0.01-0.52) | 0.36 (0.02-1.1) | 1.12 | 0.01 (0-0.02) | 0.01 (0-0.02) | -0.34 (-0.51--0.17) |
| Equatorial Guinea | 0.01 (0-0.06) | 0.04 (0-0.24) | 3 | 0.01 (0-0.04) | 0.01 (0-0.06) | 1.16 (1.02-1.3) |
| Eritrea | 0.2 (0.01-0.7) | 0.34 (0.01-1.48) | 0.7 | 0.02 (0-0.08) | 0.02 (0-0.07) | -1.39 (-1.6--1.19) |
| Estonia | 0.14 (0-0.74) | 0.24 (0-1.27) | 0.71 | 0.01 (0-0.04) | 0.01 (0-0.05) | 0.25 (-0.08-0.58) |
| Eswatini | 0.02 (0-0.1) | 0.03 (0-0.15) | 0.5 | 0.01 (0-0.04) | 0.01 (0-0.03) | -1.02 (-1.24--0.8) |
| Ethiopia | 2.58 (0.13-8.73) | 3.88 (0.09-15.26) | 0.5 | 0.02 (0-0.06) | 0.01 (0-0.04) | -1.82 (-2.07--1.56) |
| Fiji | 0.12 (0.01-0.34) | 0.24 (0.02-0.71) | 1 | 0.05 (0-0.14) | 0.04 (0-0.12) | -1.01 (-1.26--0.76) |
| Finland | 2.11 (0.01-7.96) | 2.36 (0.01-9.75) | 0.12 | 0.03 (0-0.11) | 0.02 (0-0.07) | -1.71 (-2.08--1.35) |
| France | 13.3 (0-58.59) | 14.43 (0.01-62.12) | 0.08 | 0.02 (0-0.07) | 0.01 (0-0.04) | -2.07 (-2.36--1.77) |
| Gabon | 0.07 (0-0.35) | 0.12 (0-0.63) | 0.71 | 0.01 (0-0.07) | 0.01 (0-0.08) | -0.24 (-0.44--0.04) |
| Gambia | 0.02 (0-0.13) | 0.08 (0-0.41) | 3 | 0.01 (0-0.04) | 0.01 (0-0.05) | 0.09 (-0.05-0.23) |
| Georgia | 0.33 (0.03-0.91) | 1.21 (0.05-3.83) | 2.67 | 0.01 (0-0.02) | 0.02 (0-0.06) | 6.68 (5.34-8.03) |
| Germany | 17.91 (0.1-68.23) | 24.9 (0.24-96.66) | 0.39 | 0.01 (0-0.05) | 0.01 (0-0.05) | -0.04 (-0.15-0.07) |
| Ghana | 0.75 (0-3.67) | 2.44 (0.01-10.75) | 2.25 | 0.01 (0-0.07) | 0.02 (0-0.07) | 0.44 (0.25-0.62) |
| Greece | 2.84 (0.01-11.32) | 6.33 (0.02-26.57) | 1.23 | 0.02 (0-0.07) | 0.03 (0-0.1) | 0.58 (0.34-0.82) |
| Greenland | 0.01 (0-0.03) | 0.01 (0-0.03) | 0 | 0.02 (0-0.09) | 0.01 (0-0.05) | -1.74 (-1.86--1.63) |
| Grenada | 0.03 (0-0.1) | 0.03 (0-0.11) | 0 | 0.03 (0-0.13) | 0.03 (0-0.11) | -1.86 (-2.73--0.99) |
| Guam | 0.06 (0.01-0.16) | 0.05 (0-0.14) | -0.17 | 0.11 (0.01-0.31) | 0.02 (0-0.07) | -5.11 (-5.33--4.9) |
| Guatemala | 0.21 (0.01-0.69) | 0.49 (0.02-1.5) | 1.33 | 0.01 (0-0.02) | 0 (0-0.01) | -2.03 (-2.3--1.76) |
| Guinea | 0.21 (0-1.08) | 0.41 (0-2.38) | 0.95 | 0.01 (0-0.04) | 0.01 (0-0.05) | 0.41 (0.29-0.53) |
| Guinea-Bissau | 0.03 (0-0.17) | 0.05 (0-0.26) | 0.67 | 0.01 (0-0.05) | 0.01 (0-0.05) | -0.39 (-0.47--0.3) |
| Guyana | 0.04 (0-0.16) | 0.11 (0-0.44) | 1.75 | 0.01 (0-0.05) | 0.02 (0-0.08) | 0.72 (-0.07-1.53) |
| Haiti | 0.35 (0-1.42) | 0.65 (0-2.64) | 0.86 | 0.01 (0-0.06) | 0.01 (0-0.05) | -0.78 (-0.87--0.69) |
| Honduras | 0.15 (0.01-0.49) | 0.71 (0.03-2.23) | 3.73 | 0.01 (0-0.03) | 0.01 (0-0.04) | 1.45 (1.32-1.59) |
| Hungary | 6.07 (1.35-12.18) | 8.08 (1.57-18.13) | 0.33 | 0.04 (0.01-0.09) | 0.04 (0.01-0.09) | -0.51 (-0.74--0.28) |
| Iceland | 0.09 (0-0.32) | 0.11 (0-0.4) | 0.22 | 0.03 (0-0.11) | 0.02 (0-0.06) | -2.13 (-2.46--1.79) |
| India | 19.75 (0.37-74.11) | 94.36 (3.03-337.33) | 3.78 | 0 (0-0.02) | 0.01 (0-0.03) | 2.08 (1.87-2.28) |
| Indonesia | 6.73 (1.08-16.57) | 17.71 (2-49.13) | 1.63 | 0.01 (0-0.02) | 0.01 (0-0.02) | -0.05 (-0.18-0.08) |
| Iran (Islamic Republic of) | 0.16 (0-0.92) | 0.93 (0-5.09) | 4.81 | 0 (0-0) | 0 (0-0.01) | 2.91 (2.51-3.31) |
| Iraq | 0.05 (0-0.29) | 0.18 (0-1.01) | 2.6 | 0 (0-0) | 0 (0-0) | 0.29 (0.21-0.37) |
| Ireland | 0.89 (0-4.15) | 0.81 (0-3.95) | -0.09 | 0.02 (0-0.1) | 0.01 (0-0.05) | -2.62 (-2.88--2.35) |
| Israel | 0.51 (0-2.12) | 0.83 (0-3.52) | 0.63 | 0.01 (0-0.04) | 0.01 (0-0.03) | -2.09 (-2.31--1.86) |
| Italy | 16.26 (0.92-51.6) | 25.52 (0.65-94.49) | 0.57 | 0.02 (0-0.06) | 0.02 (0-0.06) | -0.73 (-1.02--0.44) |
| Jamaica | 0.23 (0-0.91) | 0.34 (0-1.37) | 0.48 | 0.01 (0-0.05) | 0.01 (0-0.04) | -0.78 (-1.13--0.42) |
| Japan | 64.01 (10.82-149.75) | 240.86 (12.92-733.85) | 2.76 | 0.04 (0.01-0.09) | 0.05 (0-0.16) | 0.99 (0.8-1.19) |
| Jordan | 0.04 (0-0.2) | 0.17 (0-0.99) | 3.25 | 0 (0-0.02) | 0 (0-0.01) | -0.62 (-0.83--0.41) |
| Kazakhstan | 1.74 (0.14-4.98) | 2.36 (0.08-8.25) | 0.36 | 0.01 (0-0.04) | 0.01 (0-0.05) | -1.03 (-1.39--0.67) |
| Kenya | 0.53 (0.02-1.95) | 1.19 (0.04-4.68) | 1.25 | 0.01 (0-0.03) | 0.01 (0-0.03) | -0.46 (-0.65--0.28) |
| Kiribati | 0 (0-0) | 0 (0-0.01) | NA | 0.01 (0-0.02) | 0.01 (0-0.02) | -0.31 (-0.44--0.18) |
| Kuwait | 0.03 (0-0.14) | 0.13 (0-0.54) | 3.33 | 0 (0-0.02) | 0 (0-0.02) | 0.37 (-0.73-1.49) |
| Kyrgyzstan | 0.1 (0.01-0.3) | 0.32 (0.01-1.08) | 2.2 | 0 (0-0.01) | 0.01 (0-0.02) | 2.98 (2.34-3.62) |
| Lao People's Democratic Republic | 0.25 (0.04-0.63) | 0.5 (0.04-1.46) | 1 | 0.01 (0-0.04) | 0.01 (0-0.04) | -0.39 (-0.48--0.3) |
| Latvia | 0.46 (0.01-1.69) | 0.68 (0.01-2.44) | 0.48 | 0.01 (0-0.05) | 0.02 (0-0.06) | 0.38 (0.05-0.7) |
| Lebanon | 0.15 (0-0.96) | 0.3 (0-1.76) | 1 | 0.01 (0-0.05) | 0 (0-0.03) | -1 (-1.25--0.74) |
| Lesotho | 0.04 (0-0.22) | 0.05 (0-0.28) | 0.25 | 0.01 (0-0.03) | 0.01 (0-0.03) | 0.31 (0.14-0.48) |
| Liberia | 0.09 (0-0.49) | 0.13 (0-0.73) | 0.44 | 0.01 (0-0.05) | 0.01 (0-0.04) | -0.32 (-0.46--0.18) |
| Libya | 0.01 (0-0.03) | 0.04 (0-0.22) | 3 | 0 (0-0) | 0 (0-0) | 3.94 (3.56-4.31) |
| Lithuania | 0.42 (0-1.79) | 0.76 (0-3.28) | 0.81 | 0.01 (0-0.04) | 0.01 (0-0.06) | 0.88 (0.65-1.1) |
| Luxembourg | 0.11 (0-0.47) | 0.12 (0-0.5) | 0.09 | 0.02 (0-0.09) | 0.01 (0-0.04) | -2.52 (-2.72--2.32) |
| Madagascar | 1.67 (0.08-5.7) | 1.69 (0.03-6.44) | 0.01 | 0.04 (0-0.13) | 0.02 (0-0.08) | -2.42 (-2.65--2.19) |
| Malawi | 0.52 (0.02-1.84) | 0.82 (0.01-3.23) | 0.58 | 0.02 (0-0.06) | 0.01 (0-0.05) | -1.13 (-1.34--0.92) |
| Malaysia | 4.63 (0.71-11.1) | 11.74 (1.37-30.34) | 1.54 | 0.06 (0.01-0.14) | 0.05 (0.01-0.12) | -1.64 (-2.1--1.17) |
| Maldives | 0.01 (0-0.02) | 0.02 (0-0.07) | 1 | 0.01 (0-0.03) | 0.01 (0-0.02) | -2.01 (-2.2--1.82) |
| Mali | 0.16 (0-0.79) | 0.36 (0-2.05) | 1.25 | 0 (0-0.02) | 0 (0-0.03) | 0.09 (-0.11-0.29) |
| Malta | 0.06 (0-0.24) | 0.09 (0-0.35) | 0.5 | 0.02 (0-0.06) | 0.01 (0-0.03) | -2.01 (-2.33--1.69) |
| Marshall Islands | 0.01 (0-0.02) | 0.01 (0-0.03) | 0 | 0.05 (0-0.13) | 0.04 (0-0.12) | -0.84 (-0.93--0.76) |
| Mauritania | 0.1 (0-0.48) | 0.18 (0-1.01) | 0.8 | 0.01 (0-0.05) | 0.01 (0-0.05) | -0.65 (-0.81--0.49) |
| Mauritius | 0.17 (0.03-0.42) | 0.22 (0.03-0.58) | 0.29 | 0.03 (0-0.07) | 0.01 (0-0.03) | -2.5 (-3.04--1.97) |
| Mexico | 2.14 (0.04-7.87) | 5.15 (0.08-18.46) | 1.41 | 0.01 (0-0.02) | 0 (0-0.02) | -1.44 (-1.63--1.24) |
| Micronesia (Federated States of) | 0.02 (0-0.07) | 0.02 (0-0.07) | 0 | 0.06 (0-0.19) | 0.05 (0-0.14) | -1.2 (-1.33--1.07) |
| Monaco | 0.02 (0-0.09) | 0.03 (0-0.12) | 0.5 | 0.02 (0-0.11) | 0.02 (0-0.1) | 0.14 (0.01-0.27) |
| Mongolia | 0.03 (0-0.1) | 0.1 (0-0.37) | 2.33 | 0 (0-0.01) | 0 (0-0.02) | 1.27 (0.82-1.72) |
| Montenegro | 0.76 (0.14-1.76) | 1.31 (0.23-3.08) | 0.72 | 0.12 (0.02-0.29) | 0.14 (0.02-0.33) | 0.52 (0.29-0.75) |
| Morocco | 0.04 (0-0.24) | 0.29 (0-1.57) | 6.25 | 0 (0-0) | 0 (0-0) | 4.43 (4.18-4.67) |
| Mozambique | 1 (0.04-3.43) | 1.53 (0.02-5.98) | 0.53 | 0.02 (0-0.07) | 0.02 (0-0.06) | -0.64 (-0.73--0.55) |
| Myanmar | 2.63 (0.35-6.4) | 5.41 (0.56-14.54) | 1.06 | 0.01 (0-0.03) | 0.01 (0-0.04) | -0.27 (-0.39--0.16) |
| Namibia | 0.05 (0-0.22) | 0.08 (0-0.39) | 0.6 | 0.01 (0-0.04) | 0.01 (0-0.03) | -1 (-1.17--0.83) |
| Nauru | 0 (0-0.01) | 0 (0-0.01) | NA | 0.06 (0.01-0.17) | 0.06 (0-0.21) | -0.06 (-0.12--0.01) |
| Nepal | 0.27 (0-1.27) | 1.96 (0.01-7.65) | 6.26 | 0 (0-0.02) | 0.01 (0-0.04) | 3.89 (3.62-4.16) |
| Netherlands | 5.9 (0-28.87) | 6.26 (0.01-27.59) | 0.06 | 0.03 (0-0.14) | 0.02 (0-0.07) | -2.08 (-2.39--1.76) |
| New Zealand | 1.71 (0-7.29) | 1.75 (0-7.8) | 0.02 | 0.04 (0-0.18) | 0.02 (0-0.09) | -2.99 (-3.23--2.76) |
| Nicaragua | 0.05 (0-0.16) | 0.17 (0.01-0.53) | 2.4 | 0 (0-0.01) | 0 (0-0.01) | -0.05 (-0.28-0.18) |
| Niger | 0.11 (0-0.57) | 0.31 (0-1.66) | 1.82 | 0.01 (0-0.03) | 0 (0-0.03) | -0.33 (-0.41--0.25) |
| Nigeria | 3.56 (0-20.21) | 4.91 (0-26.79) | 0.38 | 0.01 (0-0.05) | 0.01 (0-0.04) | -1.92 (-2.21--1.62) |
| Niue | 0 (0-0) | 0 (0-0) | NA | 0.04 (0-0.13) | 0.03 (0-0.1) | -1.23 (-1.34--1.12) |
| North Macedonia | 0.65 (0.14-1.46) | 1.34 (0.21-3.53) | 1.06 | 0.04 (0.01-0.08) | 0.04 (0.01-0.11) | 0.22 (0.02-0.42) |
| Northern Mariana Islands | 0.01 (0-0.02) | 0.02 (0-0.05) | 1 | 0.08 (0.01-0.23) | 0.04 (0-0.13) | -3.08 (-3.39--2.76) |
| Norway | 2 (0.01-8.27) | 2.09 (0.01-8.58) | 0.04 | 0.03 (0-0.11) | 0.02 (0-0.08) | -1.57 (-1.97--1.17) |
| Oman | 0 (0-0.02) | 0.03 (0-0.18) | Inf | 0 (0-0) | 0 (0-0.01) | 4.04 (3.59-4.5) |
| Pakistan | 2.09 (0-8.96) | 10.01 (0.13-37.4) | 3.79 | 0 (0-0.02) | 0.01 (0-0.04) | 3.17 (2.78-3.57) |
| Palau | 0 (0-0.01) | 0.01 (0-0.02) | Inf | 0.05 (0-0.15) | 0.04 (0-0.12) | -1.19 (-1.26--1.12) |
| Palestine | 0.01 (0-0.08) | 0.04 (0-0.27) | 3 | 0 (0-0.01) | 0 (0-0.01) | 0.65 (0.55-0.74) |
| Panama | 0.35 (0.02-1.05) | 0.81 (0.04-2.51) | 1.31 | 0.02 (0-0.07) | 0.02 (0-0.06) | -1.5 (-1.72--1.29) |
| Papua New Guinea | 0.28 (0.02-0.81) | 0.79 (0.05-2.49) | 1.82 | 0.02 (0-0.06) | 0.02 (0-0.07) | -0.32 (-0.44--0.21) |
| Paraguay | 0.43 (0.01-1.41) | 1.49 (0.03-5.04) | 2.47 | 0.02 (0-0.07) | 0.03 (0-0.09) | 0.87 (0.77-0.97) |
| Peru | 0.93 (0.02-3.1) | 2.01 (0.04-7.1) | 1.16 | 0.01 (0-0.03) | 0.01 (0-0.02) | -1.42 (-1.62--1.22) |
| Philippines | 5.15 (0.79-13.09) | 14.16 (1.65-39.46) | 1.75 | 0.02 (0-0.06) | 0.02 (0-0.06) | -0.32 (-0.41--0.24) |
| Poland | 24.66 (2.82-65.25) | 36.52 (4.15-99.62) | 0.48 | 0.06 (0.01-0.15) | 0.05 (0.01-0.13) | -0.84 (-1.05--0.63) |
| Portugal | 0.9 (0-4.15) | 2.25 (0.03-9.09) | 1.5 | 0.01 (0-0.03) | 0.01 (0-0.04) | 1.16 (0.98-1.34) |
| Puerto Rico | 0.56 (0-2.1) | 0.46 (0-1.88) | -0.18 | 0.02 (0-0.06) | 0.01 (0-0.02) | -4.38 (-4.66--4.1) |
| Qatar | 0 (0-0.02) | 0.02 (0-0.13) | Inf | 0 (0-0.02) | 0 (0-0.01) | -1.78 (-2.27--1.29) |
| Republic of Korea | 7.36 (0.75-20.04) | 35.76 (4.39-95.06) | 3.86 | 0.03 (0-0.08) | 0.04 (0-0.1) | 1.02 (0.7-1.33) |
| Republic of Moldova | 0.16 (0-0.69) | 0.3 (0-1.4) | 0.87 | 0 (0-0.02) | 0.01 (0-0.02) | 0.69 (0.39-0.99) |
| Romania | 5.73 (1.18-12.44) | 9.85 (1.63-23.41) | 0.72 | 0.02 (0-0.05) | 0.03 (0-0.06) | 0.54 (0.33-0.74) |
| Russian Federation | 38.64 (2.18-120.03) | 80.47 (3.97-251.35) | 1.08 | 0.02 (0-0.07) | 0.03 (0-0.11) | 1.17 (0.77-1.57) |
| Rwanda | 0.8 (0.02-2.7) | 0.83 (0.01-3.19) | 0.04 | 0.04 (0-0.12) | 0.02 (0-0.07) | -3.15 (-3.54--2.76) |
| Saint Kitts and Nevis | 0.01 (0-0.04) | 0.01 (0-0.03) | 0 | 0.02 (0-0.09) | 0.01 (0-0.06) | -2.13 (-2.39--1.88) |
| Saint Lucia | 0.04 (0-0.17) | 0.06 (0-0.24) | 0.5 | 0.06 (0-0.23) | 0.03 (0-0.1) | -4.06 (-4.5--3.62) |
| Saint Vincent and the Grenadines | 0.01 (0-0.05) | 0.02 (0-0.07) | 1 | 0.02 (0-0.07) | 0.01 (0-0.05) | -1.82 (-2.12--1.52) |
| Samoa | 0.01 (0-0.04) | 0.02 (0-0.07) | 1 | 0.01 (0-0.06) | 0.02 (0-0.07) | -0.16 (-0.36-0.04) |
| San Marino | 0.01 (0-0.02) | 0.01 (0-0.04) | 0 | 0.02 (0-0.07) | 0.01 (0-0.04) | -0.41 (-0.84-0.01) |
| Sao Tome and Principe | 0 (0-0.02) | 0.01 (0-0.04) | Inf | 0.01 (0-0.03) | 0.01 (0-0.04) | 0.97 (0.9-1.03) |
| Saudi Arabia | 0.01 (0-0.05) | 0.09 (0-0.49) | 8 | 0 (0-0) | 0 (0-0) | 3.8 (3.36-4.25) |
| Senegal | 0.2 (0-1.07) | 0.51 (0-2.74) | 1.55 | 0.01 (0-0.04) | 0.01 (0-0.04) | -0.11 (-0.27-0.06) |
| Serbia | 5.87 (1.18-13.05) | 10.41 (1.96-23.7) | 0.77 | 0.06 (0.01-0.13) | 0.06 (0.01-0.14) | 0.23 (0.12-0.34) |
| Seychelles | 0.01 (0-0.02) | 0.01 (0-0.04) | 0 | 0.02 (0-0.04) | 0.01 (0-0.04) | -0.8 (-0.96--0.65) |
| Sierra Leone | 0.11 (0-0.57) | 0.19 (0-1.02) | 0.73 | 0.01 (0-0.03) | 0.01 (0-0.03) | 0.18 (0.12-0.23) |
| Singapore | 0.91 (0.11-2.39) | 2.93 (0.21-8.03) | 2.22 | 0.05 (0.01-0.12) | 0.04 (0-0.1) | -0.66 (-0.9--0.42) |
| Slovakia | 2.58 (0.56-5.77) | 3.52 (0.62-8.18) | 0.36 | 0.04 (0.01-0.1) | 0.04 (0.01-0.09) | -0.56 (-0.62--0.5) |
| Slovenia | 1.24 (0.25-2.63) | 1.65 (0.3-3.8) | 0.33 | 0.05 (0.01-0.11) | 0.03 (0.01-0.08) | -1.55 (-1.82--1.29) |
| Solomon Islands | 0.02 (0-0.07) | 0.06 (0-0.19) | 2 | 0.03 (0-0.08) | 0.03 (0-0.08) | -0.39 (-0.52--0.26) |
| Somalia | 0.28 (0.01-0.99) | 0.35 (0-1.35) | 0.25 | 0.02 (0-0.06) | 0.01 (0-0.03) | -2.71 (-2.9--2.51) |
| South Africa | 1.6 (0-7.72) | 2.03 (0-10.97) | 0.27 | 0.01 (0-0.04) | 0 (0-0.03) | -2.37 (-2.7--2.05) |
| South Sudan | 0.59 (0.02-2.14) | 0.34 (0-1.35) | -0.42 | 0.03 (0-0.09) | 0.01 (0-0.05) | -3.08 (-3.33--2.82) |
| Spain | 3.38 (0-17.7) | 6.11 (0.04-30.84) | 0.81 | 0.01 (0-0.03) | 0.01 (0-0.03) | -0.38 (-0.67--0.09) |
| Sri Lanka | 0.55 (0.08-1.34) | 1.21 (0.1-3.21) | 1.2 | 0.01 (0-0.02) | 0 (0-0.01) | -0.59 (-0.74--0.44) |
| Sudan | 0.02 (0-0.15) | 0.14 (0-0.74) | 6 | 0 (0-0) | 0 (0-0) | 3.95 (3.73-4.17) |
| Suriname | 0.04 (0-0.16) | 0.08 (0-0.32) | 1 | 0.02 (0-0.07) | 0.01 (0-0.05) | -1.02 (-1.21--0.83) |
| Sweden | 5.83 (0.03-22.65) | 4.87 (0.01-19.9) | -0.16 | 0.04 (0-0.14) | 0.02 (0-0.08) | -2.19 (-2.52--1.86) |
| Switzerland | 3.02 (0.01-12.71) | 2.66 (0.01-10.98) | -0.12 | 0.03 (0-0.12) | 0.01 (0-0.05) | -2.46 (-2.64--2.28) |
| Syrian Arab Republic | 0.04 (0-0.25) | 0.12 (0-0.71) | 2 | 0 (0-0) | 0 (0-0.01) | 0.39 (0.31-0.48) |
| Taiwan | 2.41 (0.13-6.97) | 11.37 (0.6-34.72) | 3.72 | 0.02 (0-0.05) | 0.03 (0-0.08) | 0.33 (-0.4-1.07) |
| Tajikistan | 0.1 (0.01-0.28) | 0.11 (0-0.37) | 0.1 | 0 (0-0.01) | 0 (0-0.01) | -1.91 (-2.17--1.65) |
| Thailand | 10.56 (1.34-27.56) | 32.78 (3.2-93.42) | 2.1 | 0.04 (0-0.1) | 0.03 (0-0.09) | -1.34 (-1.52--1.15) |
| Timor-Leste | 0.02 (0-0.05) | 0.07 (0.01-0.2) | 2.5 | 0.01 (0-0.02) | 0.01 (0-0.03) | -0.07 (-0.2-0.06) |
| Togo | 0.08 (0-0.43) | 0.27 (0-1.43) | 2.38 | 0.01 (0-0.04) | 0.01 (0-0.04) | -0.22 (-0.34--0.11) |
| Tokelau | 0 (0-0) | 0 (0-0) | NA | 0.05 (0-0.15) | 0.04 (0-0.13) | -0.98 (-1.13--0.82) |
| Tonga | 0.02 (0-0.05) | 0.03 (0-0.09) | 0.5 | 0.04 (0-0.12) | 0.04 (0-0.12) | -0.61 (-0.83--0.38) |
| Trinidad and Tobago | 0.2 (0-0.83) | 0.34 (0-1.4) | 0.7 | 0.03 (0-0.11) | 0.02 (0-0.07) | -1.68 (-1.93--1.42) |
| Tunisia | 0.02 (0-0.12) | 0.13 (0-0.79) | 5.5 | 0 (0-0) | 0 (0-0.01) | 3.64 (3.33-3.95) |
| T眉rkiye | 1.2 (0-8.1) | 3.57 (0-23.83) | 1.98 | 0 (0-0.02) | 0 (0-0.03) | 0.34 (0.02-0.67) |
| Turkmenistan | 0.21 (0.02-0.63) | 0.53 (0.02-1.87) | 1.52 | 0.01 (0-0.04) | 0.01 (0-0.05) | 0.19 (-0.03-0.42) |
| Tuvalu | 0 (0-0.01) | 0 (0-0.01) | NA | 0.04 (0-0.14) | 0.04 (0-0.11) | -0.89 (-0.99--0.78) |
| Uganda | 0.91 (0.03-3.15) | 1.43 (0.02-5.4) | 0.57 | 0.02 (0-0.06) | 0.01 (0-0.05) | -1.24 (-1.5--0.99) |
| Ukraine | 6.41 (0.01-26.37) | 7.73 (0.01-36.37) | 0.21 | 0.01 (0-0.04) | 0.01 (0-0.05) | -0.08 (-0.48-0.31) |
| United Arab Emirates | 0.01 (0-0.08) | 0.11 (0-0.6) | 10 | 0 (0-0.02) | 0 (0-0.02) | 2.13 (1.39-2.87) |
| United Kingdom | 28.09 (0.09-117.86) | 28.59 (0.16-122.52) | 0.02 | 0.03 (0-0.12) | 0.02 (0-0.08) | -1.3 (-1.77--0.83) |
| United Republic of Tanzania | 2.91 (0.17-8.79) | 4.59 (0.2-15.7) | 0.58 | 0.03 (0-0.1) | 0.02 (0-0.07) | -2.12 (-2.34--1.89) |
| United States of America | 63.63 (0.04-293.04) | 81.95 (1.07-313.2) | 0.29 | 0.02 (0-0.09) | 0.01 (0-0.05) | -1.25 (-1.67--0.84) |
| United States Virgin Islands | 0.01 (0-0.06) | 0.02 (0-0.09) | 1 | 0.02 (0-0.08) | 0.01 (0-0.05) | -2.28 (-2.51--2.04) |
| Uruguay | 2.38 (0.05-7.64) | 2.44 (0.06-8.17) | 0.03 | 0.06 (0-0.19) | 0.04 (0-0.14) | -1.62 (-1.88--1.36) |
| Uzbekistan | 0.29 (0.02-0.95) | 1.93 (0.07-6.94) | 5.66 | 0 (0-0.01) | 0.01 (0-0.03) | 4.46 (3.54-5.39) |
| Vanuatu | 0.01 (0-0.03) | 0.03 (0-0.08) | 2 | 0.02 (0-0.06) | 0.02 (0-0.06) | -0.62 (-0.7--0.53) |
| Venezuela (Bolivarian Republic of) | 1.87 (0.1-5.56) | 5.01 (0.24-15.37) | 1.68 | 0.02 (0-0.06) | 0.02 (0-0.05) | -1.11 (-1.43--0.8) |
| Viet Nam | 4.18 (0.54-10.55) | 11.42 (1.01-31.19) | 1.73 | 0.01 (0-0.03) | 0.01 (0-0.04) | 0.09 (-0.07-0.25) |
| Yemen | 0.01 (0-0.07) | 0.11 (0-0.59) | 10 | 0 (0-0) | 0 (0-0) | 4.23 (3.86-4.61) |
| Zambia | 0.66 (0.03-2.15) | 1.63 (0.02-6.92) | 1.47 | 0.03 (0-0.1) | 0.03 (0-0.13) | -0.05 (-0.43-0.33) |
| Zimbabwe | 0.47 (0-2.07) | 0.91 (0-3.87) | 0.94 | 0.01 (0-0.06) | 0.02 (0-0.07) | 0.1 (-0.09-0.3) |

Supplementary table 2. The deaths of aortic aneurysm attributable to diet high in sodium cases and rates in 1990 and 2021 across 204 countries, and the trends from 1990 to 2021.
