# Supplementary material for: Global burden of aortic aneurysm attributable to high-sodium diet from 1990 to 2021
Source: Front Nutr. 2025 Aug 21;12:1653773. doi: 10.3389/fnut.2025.1653773 (PMC12408317; doi:10.3389/fnut.2025.1653773)
Supplement: Supplementary file 2 [file Image_1.pdf]

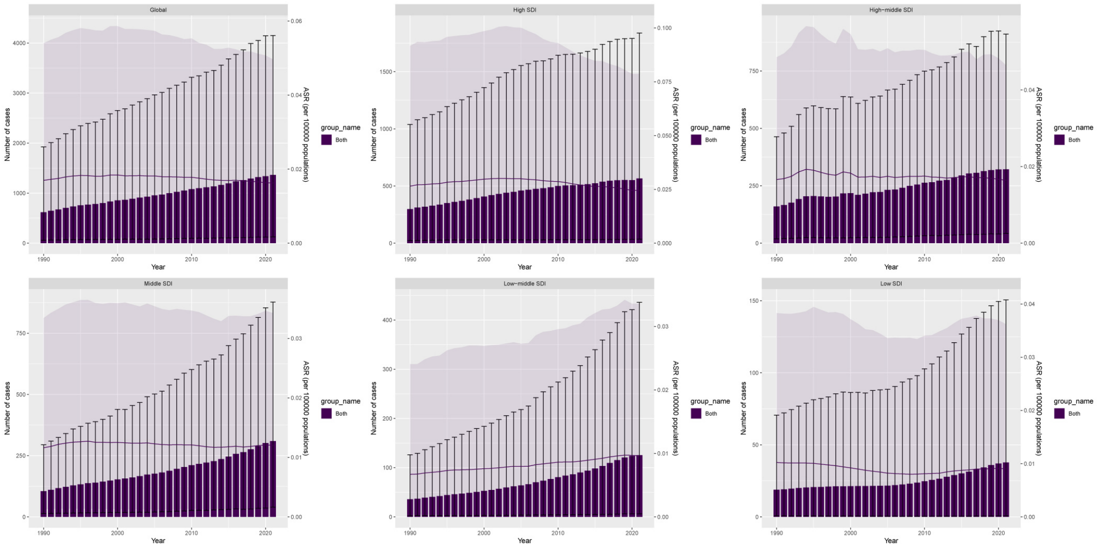

Supplementary figure 1. Death cases and ASMR of aortic aneurysm attributable to diet high in sodium from 1990 to 2021.
